# Modeling left ventricular dynamics with characteristic deformation modes

**DOI:** 10.1007/s10237-019-01168-8

**Published:** 2019-05-25

**Authors:** Brian D. Hong, Michael J. Moulton, Timothy W. Secomb

**Affiliations:** 1 Department of Surgery, University of Nebraska Medical Center, Omaha, NE USA; 2 Program in Applied Mathematics, University of Arizona, Tucson, AZ USA; 3 Department of Physiology, University of Arizona, Tucson, AZ USA

**Keywords:** Left ventricle, Computational modeling, Cardiac mechanics, 3D simulation, Deformation modes

## Abstract

A computationally efficient method is described for simulating the dynamics of the left ventricle (LV) in three dimensions. LV motion is represented as a combination of a limited number of deformation modes, chosen to represent observed cardiac motions while conserving volume in the LV wall. The contribution of each mode to wall motion is determined by a corresponding time-dependent deformation variable. The principle of virtual work is applied to these deformation variables, yielding a system of ordinary differential equations for LV dynamics, including effects of muscle fiber orientations, active and passive stresses, and surface tractions. Passive stress is governed by a transversely isotropic elastic model. Active stress acts in the fiber direction and incorporates length–tension and force–velocity properties of cardiac muscle. Preload and afterload are represented by lumped vascular models. The variational equations and their numerical solutions are verified by comparison to analytic solutions of the strong form equations. Deformation modes are constructed using Fourier series with an arbitrary number of terms. Greater numbers of deformation modes increase deformable model resolution but at increased computational cost. Simulations of normal LV motion throughout the cardiac cycle are presented using models with 8, 23, or 46 deformation modes. Aggregate quantities that describe LV function vary little as the number of deformation modes is increased. Spatial distributions of stress and strain change as more deformation modes are included, but overall patterns are conserved. This approach yields three-dimensional simulations of the cardiac cycle on a clinically relevant time-scale.

**Electronic supplementary material:**

The online version of this article (10.1007/s10237-019-01168-8) contains supplementary material, which is available to authorized users.

## Introduction

The mechanical pumping performance of the left ventricle (LV) depends in a complex way on the ventricular geometry, the passive mechanical properties of the myocardium, the arrangement of cardiac muscle fibers, and the fibers’ contractile force generation. Quantitative understanding of the effects of these characteristics on ventricular function requires the use of theoretical models to simulate the dynamics of the LV. Such models typically employ a continuum mechanics approach in which the active and passive components of stress at each point in the myocardium are expressed as time-dependent functions of local myocardial strain. The equations of equilibrium of mechanical stresses are then solved using approximate methods, subject to boundary conditions that include the external forces acting on the tissue.

Three-dimensional models of the LV are typically developed through the application of the finite element method (FEM) (Nash and Hunter 2000; Costa et al. 2001; Kerckhoffs et al. 2005). In this approach, the integrals of the variational equations (15) are split into integrals over local elements, and the displacement variations $\delta u$ are given in terms of the local element displacement functions (Zienkiewicz et al. 2005). The resulting large sparse matrix systems may be solved for the local element displacements. FEM simulations of LV dynamics yield detailed descriptions of cardiac deformation and stress. However, such models are computationally demanding due to the many degrees of freedom (DOF) that are needed to describe local element displacements.

While the FEM approach is suitable for many purposes, some problems do not require so many DOF. For example, Nordbø et al. (2014) showed that, despite their use of an elastic FEM model with hundreds of degrees of freedom, the elasticity parameters of a mouse LV were identified with greater certainty using an objective that included only four aggregated quantities: LV long-axis length, short-axis diameter, work, and volume. In such cases, a reduced model of LV kinematics would likely be sufficient and afford better parameter identifiability. Arts et al. (1992) demonstrated that 13 kinematic parameters were sufficient to fit 14 markers recorded in a canine LV model, suggesting that, for studies where only limited data is recorded, a reduced approach is suitable. Simulations of cardiac remodeling are another area where a reduced model would offer an efficient alternative to FEM modeling. The results of a reduced model would likely be similar to those found using an FEM model, as changes to cardiac function based on remodeling are distributed over the myocardium.

The continued prevalence of heart failure (Benjamin et al. 2018; Kapoor et al. 2016; Kovács 2015), among other cardiac pathologies, has lead to increasing interest in improved methods for individualized quantification of cardiac function. Patient-specific computational models of the heart offer information beyond standard clinical indices. While FEM models have been effectively applied to estimate cardiac mechanics for specific geometries (Aguado-Sierra et al. 2011; Krishnamurthy et al. 2013), their theoretical and computational complexity impedes their widespread use. A simplified method that can still represent the 3D geometry and essential deformable characteristics of the LV would provide a more accessible method for use outside the modeling community.

To these ends, we develop a computationally efficient variational method for modeling LV dynamics in Sect. 2. We have previously described this method for an axisymmetric geometry (Moulton et al. 2017) and here extend it to more general geometries and kinematics. The equation of virtual work (15) is used to describe the dynamics of the LV. However, rather than splitting the integrals and displacements into local elements as in the FEM, we describe displacements according to a set of kinematic variables $\varvec{q}$ that extend over the entire myocardial domain. The displacement variations $\delta \varvec{u}$ are therefore described in terms of variations of these kinematic variables.

We define a non-axisymmetric unstressed myocardial domain $\varOmega _0$ in prolate spheroidal coordinates. To simulate LV dynamics, we construct a mechanical model that incorporates muscle fiber orientations, active and passive stresses, and surface tractions. We assume the standard transversely isotropic elastic model. To represent the viscoelastic properties of the myocardium, the passive stress also includes a viscous component. The active stress acts only in the fiber direction and incorporates important fiber properties such as the length–tension and force–velocity relationships. These definitions are introduced into the virtual work equation, yielding a system of ordinary differential equations (ODEs) that characterize the time-dependent deformable mechanics of the LV. The validity of the method is evaluated by comparison with analytical solutions to the strong form equations. Simulations of normal cardiac function are presented and cardiac muscle fiber stress distributions are computed, demonstrating the ability of this approach to describe spatial variations in stress.

## Methods

Prolate spheroidal coordinates $(\mu ,\nu ,\phi )$, which provide a natural framework for modeling the left ventricle (Sandler and Dodge 1968; Wu et al. 2013; Heyde et al. 2016), are related to Cartesian coordinates (*x*, *y*, *z*) through

**Fig. 1:**
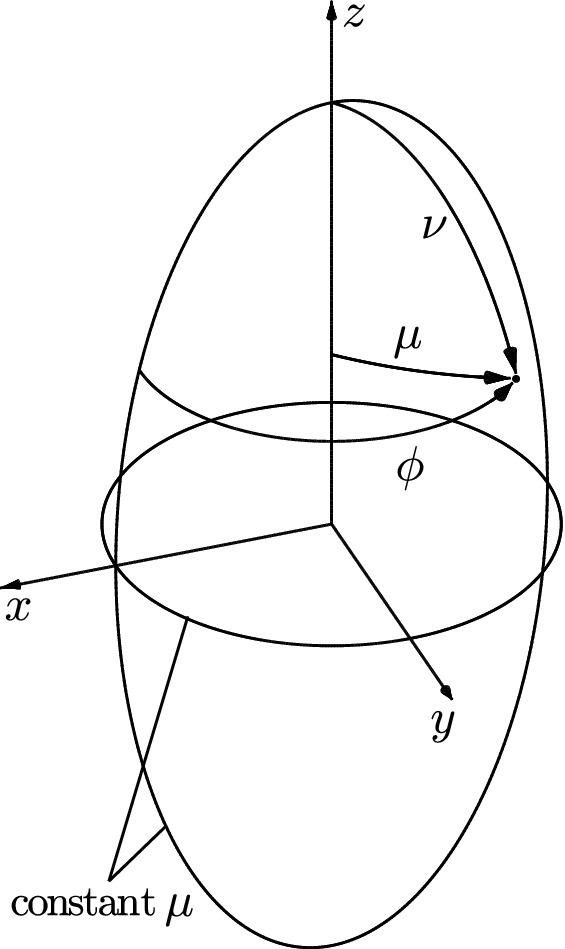
Prolate spheroidal coordinates

1 $$\begin{aligned} \begin{aligned} x&= a \sinh \mu \sin \nu \cos \phi \\ y&= a \sinh \mu \sin \nu \sin \phi \\ z&= a \cosh \mu \cos \nu , \end{aligned} \end{aligned}$$

where $\phi \in [0, 2 \pi )$ is the polar angle, $\mu \in [0, \infty )$ defines the extension out from the ellipsoid axis (analogous to the radial coordinate in spherical coordinates), $\nu \in [0, \pi ]$ is comparable to the azimuthal angle, and *a* defines the focal length of the ellipse. These coordinates are illustrated in Fig. 1. Corresponding coordinates $(x_0,y_0,z_0)$ and $(\mu _0,\nu _0,\phi _0)$ describe the reference configuration.

### Myocardial domain

We define the myocardial domain in the reference configuration in terms of its endocardial, epicardial, and basal boundaries. The endocardial and epicardial surfaces are given by $\mu _\mathrm{in0}(\nu _0,\phi _0)$ and $\mu _\mathrm{out0}(\nu _0,\phi _0)$, respectively. We define each surface as a set of bicubic splines:

2 $$\begin{aligned} \begin{aligned}&\mu _\mathrm{in0} = f(\nu _0,\phi _0; \,\varvec{c}_\mathrm{in0} ) \\&\mu _\mathrm{out0} = f(\nu _0,\phi _0;\, \varvec{c}_\mathrm{out0} ) , \end{aligned} \end{aligned}$$

where $\varvec{c}_\mathrm{in0}$ and $\varvec{c}_\mathrm{out0}$ denote sets of parameters.

The formulation of the bicubic spline functions *f* in prolate spheroidal coordinates is described in the supplementary material Section S2. We also define a function $\nu _\mathrm{up0}(\phi _0)$ that describes the basal boundary. We compute $\nu _\mathrm{up0}(\phi _0)$ by a one-dimensional periodic spline function. Thus, the reference myocardial domain is

3 $$\begin{aligned} \begin{aligned}&\varOmega _0 = \{ (\mu _0, \nu _0, \phi _0) : \mu _\mathrm{in0}(\nu _0,\phi _0) \le \mu _0 \le \mu _\mathrm{out0}(\nu _0,\phi _0), \\& \nu _\mathrm{up0}(\phi _0) \le \nu _0 \le \pi , 0 \le \phi _0 < 2\pi \}. \end{aligned} \end{aligned}$$

**Fig. 2:**
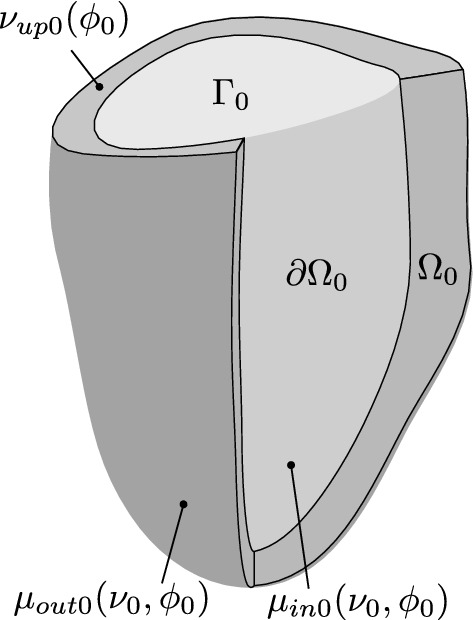
Diagram of the LV model. The myocardial reference domain $\varOmega _0$ is determined by the bounding surfaces $\mu _\mathrm{in0}$, $\mu _\mathrm{out0}$, and $\nu _\mathrm{up0}$. For the purposes of integrating the virtual work equations (21), the myocardial boundary $\partial \varOmega _0$ only includes the endocardial surface, as the other surfaces are assumed to be tractionless. The LV cavity is closed by an additional surface $\varGamma _0$

The myocardial domain and bounding surfaces are illustrated in Fig. 2.

**Fig. 3:**
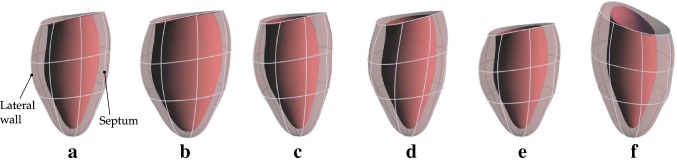
Examples of deformation according to single modes defined using the displacement functions (10) with isolated terms of (11). **a** Reference shape, **b** uniform expansion, **c** asymmetric $\mu$ deformation which expands the lateral wall but not the septum, **d** uniform torsion, **e** uniform shortening, **f** asymmetric $\nu$ deformation which lengthens the lateral wall while shortening the septum

### Kinematics

The myocardium may be deformed by an arbitrary displacement from reference coordinates $(\mu _0, \nu _0, \phi _0)$ to deformed coordinates $(\mu , \nu , \phi )$. The deformed myocardial domain $\varOmega$ is the image of the reference domain $\varOmega _0$ under such a mapping. In this section, we construct a mapping that describes LV deformations using a limited number of deformation modes. The contribution of these modes to LV displacement is determined by kinematic variables $q_i$. We evaluate the ability of these modes to represent actual cardiac deformations by analysis of tagged cardiac MRI data in Sect. 2.2.4.

#### Incompressible framework

In this section, we show how three-dimensional volume preserving deformations of the LV wall can be derived, given specified displacements of the endocardial surface. It is emphasized that this analysis is independent of the choice of functions used to describe the shape and deformation. The constraint of incompressibility is directly imposed on the deformation modes. While this assumption is not strictly true (May-Newman et al. 1994; Yin et al. 1996), much of the LV is nearly incompressible. Imposing this constraint avoids the need to introduce a large bulk elastic modulus, which can result in numerically challenging systems of stiff equations. We further simplify the kinematic construction by constraining the displacements in the $\nu$ and $\phi$ directions to be independent of $\mu _0$:

4 $$\begin{aligned} \phi = \phi (\nu _0, \phi _0) \text { and } \nu = \nu ( \nu _0, \phi _0 ). \end{aligned}$$

We discuss the implications of these kinematic simplifications in Sect. 4.3. Under these assumptions, the deformation gradient tensor in terms of prolate spheroidal coordinates is

5 $$\begin{aligned} \varvec{F} = \left[ \begin{array}{ccc} \dfrac{ g_\mu }{ g_{\mu _0}} \dfrac{ \partial \mu }{ \partial \mu _0 } &{} \dfrac{ g_\mu }{ g_{\nu _0}} \dfrac{ \partial \mu }{ \partial \nu _0 } &{} \dfrac{ g_\mu }{ g_{\phi _0}} \dfrac{ \partial \mu }{ \partial \phi _0 } \\ 0 &{} \dfrac{ g_\nu }{ g_{\nu _0}} \dfrac{ \partial \nu }{ \partial \nu _0 } &{} \dfrac{ g_\nu }{ g_{\phi _0}} \dfrac{ \partial \nu }{ \partial \phi _0 } \\ 0 &{} \dfrac{ g_\phi }{ g_{\nu _0}} \dfrac{ \partial \phi }{ \partial \nu _0 } &{} \dfrac{ g_\phi }{ g_{\phi _0}} \dfrac{ \partial \phi }{ \partial \phi _0 } \end{array} \right] , \end{aligned}$$

where the coordinate system scale factors are

6 $$\begin{aligned} g_\mu &= g_\nu = a \sqrt{ \sinh ^2 \mu + \sin ^2 \nu } \nonumber \\ g_\phi&= a \sinh \mu \sin \nu . \end{aligned}$$

These kinematic simplifications (4) yield a simplified form of the determinant of $\varvec{F}$, with only two nonzero terms. The incompressibility condition $\det (\mathbf {F}) = 1$ gives

7 $$\begin{aligned} \dfrac{ g_{\mu } g_{\nu } g_{\phi } }{ g_{\mu _0} g_{\nu _0} g_{\phi _0} } \dfrac{ \partial \mu }{ \partial \mu _0 } \left( \dfrac{ \partial \nu }{ \partial \nu _0 } \dfrac{ \partial \phi }{ \partial \phi _0 } - \dfrac{ \partial \nu }{ \partial \phi _0 } \dfrac{ \partial \phi }{ \partial \nu _0 } \right) = 1. \end{aligned}$$

For convenience, we set

8 $$\begin{aligned} R(\nu _0,\phi _0) = \dfrac{ \sin \nu }{ \sin \nu _0 } \left( \dfrac{ \partial \nu }{ \partial \nu _0 } \dfrac{ \partial \phi }{ \partial \phi _0 } - \dfrac{ \partial \nu }{ \partial \phi _0 } \dfrac{ \partial \phi }{ \partial \nu _0 } \right) . \end{aligned}$$

Inserting the scale factor definitions (6) into the incompressibility condition (7) and integrating in terms of $\mu _0$ yields

9 $$\begin{aligned} \begin{aligned}&a^3 R \left[ \cosh \mu \left( \frac{1}{3} \cosh ^2 \mu - \cos ^2 \nu \right) - \left( \frac{1}{3} - \cos ^2 \nu \right) \right] \\&=a^3 \left[ \cosh \mu _0 \left( \frac{1}{3} \cosh ^2 \mu _0 - \cos ^2 \nu _0 \right) - \left( \frac{1}{3} - \cos ^2 \nu _0 \right) \right] \\&- f_c( \nu _0, \phi _0 ), \end{aligned} \end{aligned}$$

where the constant of integration (in terms of $\mu$) has been chosen so that the left hand side is zero for $\mu = 0$ and the first term in the right hand side is zero if $\mu _0 = 0$. The arbitrary function $f_c(\nu _0, \phi _0)$ is chosen for consistency with displacements of the endocardial wall $\mu _\mathrm{in0}\rightarrow \mu _\mathrm{in}$ and is defined in the supplement, equation (S10). Equation (9) is a cubic in $\cosh \mu$ and can be solved analytically for $\mu$. We describe the solution in the supplementary material Section S3.

#### Deformation mode definitions

Prolate spheroidal coordinates are naturally suited to defining displacement modes that describe characteristic LV deformations. Alterations to the $\mu$ coordinate represent contraction toward or expansion away from the coordinate system axis. Changes to $\nu$ imply elongation or shortening of the LV. Displacements in the $\phi$ coordinate supply LV base-to-apex torsion. We define three functions to describe these displacements:

10 $$\begin{aligned} \begin{aligned} \mu _\mathrm{in}(\nu _0,\phi _0)&= \mu _\mathrm{in0}(\nu _0,\phi _0) + f_s(\nu _0, \phi _0; \varvec{q}_\mu ) \\ \phi (\nu _0,\phi _0)&= \phi _0 + f_s(\nu _0, \phi _0; \varvec{q}_\phi ) \\ \nu (\nu _0,\phi _0)&= \nu _0 + f_s(\nu _0, \phi _0; \varvec{q}_\nu ), \end{aligned} \end{aligned}$$

where $\varvec{q}_\mu$, $\varvec{q}_\phi$, and $\varvec{q}_\nu$ are vectors of coefficients in the displacement functions. The first function determines the displaced position of the endocardial wall $\mu _\mathrm{in}$. The $\mu$ displacement throughout the remainder of the myocardium is determined by the incompressibility condition (7). The remaining two functions directly determine changes to the $\nu$ and $\phi$ coordinates. The displacement function for each coordinate is computed as a linear combination of Fourier terms:

11 $$\begin{aligned} f_s(\nu _0,\phi _0;\, \varvec{a}, \varvec{b}) = \sum _{i,j} a_{i,j}\, f_{\nu _0}^i f_{s\phi _0}^j + \sum _{i,k} b_{i,k}\, f_{\nu _0}^i f_{c\phi _0}^k, \end{aligned}$$

where the one-dimensional basis functions are

12 $$\begin{aligned} \begin{aligned} f_{\nu _0}^i&= 1 - \cos [(i+1)(\nu _0 - \pi )] \\ f_{s \phi _0}^j&= \sin (j \phi _0) \\ f_{c \phi _0}^k&= \cos ( k\phi _0 ). \end{aligned} \end{aligned}$$

Only modified cosine modes are used in the $\nu _0$ direction, as sine functions create nonphysical deformations through the apex. The Fourier basis is a natural choice for the prolate coordinate system due to the periodicity of $\phi$. The first terms in this series (with $k = 0$) generate axisymmetric deformations. Terms with larger values of *i* develop a greater degree of local variations in the $\nu$ coordinate, while increasing values of *j* and *k* lead to a higher degree of local variations in the $\phi$ coordinate. Examples of deformations according to several modes are illustrated in Fig. 3. The total number of terms allowed for each index (*i*, *j*, *k*) determine the number of basis functions in the Fourier series, and therefore the overall deformable freedom for the coordinate under consideration ($\mu _\mathrm{in}$, $\nu$, or $\phi$).

#### Strain measures

Computation of the deformation gradient tensor $\varvec{F}$ (5) requires the derivatives of the prolate spheroidal coordinate positions with respect to the reference coordinates (supplementary material S26, S28). The right and left Cauchy–Green deformation gradient tensors are then $\varvec{C} = \varvec{F}^\top \varvec{F}$ and $\varvec{B} = \varvec{F} \varvec{F}^{\top }$. The Green and Euler-Almansi strains are

13 $$\begin{aligned} \varvec{E} = \dfrac{1}{2}(\varvec{C}-\varvec{I}) \quad \text {and} \quad \varvec{e} = \dfrac{1}{2}( \varvec{I} - \varvec{B}^{-1} ), \end{aligned}$$

where $\varvec{I}$ is the $3\times 3$ identity matrix.

#### Evaluation of the characteristic deformation mode kinematic model using cardiac MRI

As described above, we represent the kinematics of the LV with deformation modes that extend over the whole myocardium (Fig. 3). For this approach to be effective, the majority of LV deformation should be representable with relatively few modes. To illustrate that this is possible, we evaluated the ability of such modes to describe the motion of the LV observed with tagged cardiac magnetic resonance imaging (MRI) (see Figure S3). We analyzed two cases: the first from a healthy volunteer and the second from a patient with dilated cardiomyopathy. These data were previously published by Kar et al. (2014) and are used here with permission.

We estimated the deformation of the myocardium from the tagged MRI data using a deformable image registration algorithm. The myocardial walls were outlined manually in each imaging plane before the image registration was performed. The image registration method was described previously (Hong 2018) and is outlined briefly in the supplementary material Section S6. For the purposes of this kinematic evaluation, we assumed the reference configuration to be the end-systolic shape. While this is not the true reference configuration, this choice has little effect on this purely kinematic analysis.

To evaluate the ability of the kinematic model to reproduce the observed cardiac deformations, we optimized $N_q$ kinematic parameters to reduce the mean error between model displacements and registered displacements. The number of kinematic parameters $N_q$ was increased from 0 to 85. Modes were added increasing the number of terms in (11). We alternated between adding terms with increased $\nu$ and $\phi$ resolution for the $\mu$, $\nu$, and $\phi$ displacements. For each choice of $N_q$, we computed the displacement accounted for by that number of modes. The displacements were measured from end-systole to end-diastole. All rigid motion was removed from the problem, including the case where zero deformation modes were used. We computed the objective as the volume-averaged square displacement error:

14 $$\begin{aligned} O(\varvec{q}) = \dfrac{1}{V_\mathrm{m}} \int _{\varOmega _0} | \varvec{u}_\mathrm{m}( \varvec{x}_0; \varvec{q} ) - \varvec{u}_{r}(\varvec{x}_0) |^2 \ \hbox {d}V, \end{aligned}$$

where $|\cdot |$ is the Euclidean norm, $V_\mathrm{m}$ is the myocardial wall volume, $\varvec{u}_\mathrm{m}$ is the Cartesian representation of the displacement according to the selected deformation modes $\varvec{q}$, and $\varvec{u}_r$ is the displacement data registered from the cardiac MRI. The results of this analysis are shown in Sect. 3.1.

### Dynamics

For the purpose of modeling LV dynamics, gravitational and inertial effects are typically negligible (Chaudhry et al. 1997), and the equations of virtual work (Malvern 1969) may be written as:

15 $$\begin{aligned} \begin{aligned}&\int _{\varOmega } \varvec{\sigma }:\delta \varvec{e} \ \hbox {d}v - \int _{\partial \varOmega } \varvec{t} \cdot \delta \varvec{u} \ \hbox {d}s = 0. \end{aligned} \end{aligned}$$

In our approach to LV kinematics, the deformed position $\varvec{x}$ is a function of the reference position $\varvec{x}_0$ and a set of time-dependent kinematic variables: $\varvec{q} = \varvec{q}(t)$.

16 $$\begin{aligned} \varvec{x} = \varvec{x}(\varvec{x}_0, \varvec{q}) \end{aligned}$$

Each $q_i$ defines the degree of displacement according to a particular mode, described in Sect. 2.2. Consequently, the displacement vector and strain tensor are functions of the reference position and the kinematic variables:

17 $$\begin{aligned} \varvec{u} = \varvec{u}(\varvec{x}_0, \varvec{q}) \quad \text {and} \quad \varvec{e} = \varvec{e}(\varvec{x}_0, \varvec{q}). \end{aligned}$$

This implies that

18 $$\begin{aligned} \delta \varvec{u} = \sum _{i=1}^{N_q} \dfrac{\partial \varvec{u} }{ \partial q_i } \delta q_i \quad \text {and} \quad \delta \varvec{e} = \sum _{i=1}^{N_q} \dfrac{\partial \varvec{e} }{ \partial q_i } \delta q_i, \end{aligned}$$

where $N_q$ is the number of deformation modes and $\delta q_i$ is the first variation of $q_i$. The virtual work equation (15) then yields

19 $$\begin{aligned} \begin{aligned}&\sum _{i=1}^{N_q} \delta q_i \left( \int _{\varOmega } \varvec{\sigma }: \dfrac{\partial \varvec{e} }{ \partial q_i } \ \hbox {d}v - \int _{\partial \varOmega } \varvec{t} \cdot \dfrac{\partial \varvec{u} }{ \partial q_i } \ \hbox {d}s \right) = 0. \end{aligned} \end{aligned}$$

Because the variations $\delta q_i$ are arbitrary, the virtual work equation is only generally satisfied if the system of $N_q$ equations

20 $$\begin{aligned} \begin{aligned}&\int _{\varOmega } \varvec{\sigma }: \dfrac{\partial \varvec{e} }{ \partial q_i } \ \hbox {d}v - \int _{\partial \varOmega } \varvec{t} \cdot \dfrac{\partial \varvec{u} }{ \partial q_i } \ \hbox {d}s = 0 \end{aligned} \end{aligned}$$

is satisfied. The stresses and strains may also be represented and integrated in the reference domain:

21 $$\begin{aligned} \begin{aligned}&\int _{\varOmega _0} \varvec{S}: \dfrac{\partial \varvec{E} }{ \partial q_i } \ \hbox {d}V - \int _{\partial \varOmega } \varvec{t} \cdot \dfrac{\partial \varvec{u} }{ \partial q_i } \ \hbox {d}s = 0, \end{aligned} \end{aligned}$$

where $\varvec{S}$ is the second Piola–Kirchhoff (PK2) stress and $\varvec{E}$ is the Green strain. This system, together with definitions for the myocardial domain, deformation modes, stresses, and surface tractions, may be solved for either equilibrium states $\varvec{q}$ or dynamic solutions of LV motion $\varvec{q}(t)$.

#### Muscle fiber directions

The helical arrangement of cardiac muscle fibers is defined according to observations (Streeter et al. 1969; Moulton et al. 2017). Measurements using diffusion tensor MRI (Scollan et al. 1998) could also be used. An orthogonal system of local coordinates (*s*, *n*, *f*) is introduced with base vectors in the fiber direction $\varvec{e}_f$, the sheet direction $\varvec{e}_s$—which lies within the muscle sheet and is perpendicular to the fiber direction, and $\varvec{e}_n = \varvec{e}_f \times \varvec{e}_s$ (supplementary material Section S7). Quantities that are represented in terms of the local fiber coordinate basis may be transformed to the prolate spheroidal basis using an appropriate unitary rotation matrix

22 $$\begin{aligned} \varvec{Q} = \left[ \begin{array}{lll} \varvec{e}_\mu \cdot \varvec{e}_s &{} \ \varvec{e}_\mu \cdot \varvec{e}_n &{}\ \varvec{e}_\mu \cdot \varvec{e}_f \\ \varvec{e}_\nu \cdot \varvec{e}_s &{} \ \varvec{e}_\nu \cdot \varvec{e}_n &{} \ \varvec{e}_\nu \cdot \varvec{e}_f \\ \varvec{e}_\phi \cdot \varvec{e}_s &{} \ \varvec{e}_\phi \cdot \varvec{e}_n &{} \ \varvec{e}_\phi \cdot \varvec{e}_f \end{array} \right] . \end{aligned}$$

Vectors $\varvec{v}$ and tensors $\varvec{S}$ can be transformed from fiber to prolate coordinate representations by

23 $$\begin{aligned} \varvec{v}_\mathrm{prolate} = \varvec{Q} \varvec{v}_\mathrm{fiber} \quad \text {and} \quad \varvec{S}_\mathrm{prolate} = \varvec{Q} \varvec{S}_\mathrm{fiber} \varvec{Q}^{\top }. \end{aligned}$$

#### Passive elastic stress

Myocardial elasticity is represented using a transversely isotropic material law, referred to as the “Guccione law,” (Nordbø et al. 2014; Hadjicharalambous et al. 2015; Xi et al. 2011) citing work by Guccione et al. (1991). It may also be attributed to Chuong and Fung (1986). The elastic strain energy density is

24 $$\begin{aligned} \varPsi = \frac{1}{2} k_e (e^W - 1), \end{aligned}$$

where

25 $$\begin{aligned} \begin{aligned} e^W &= \exp [b_{ff} E_{ff}^2 + b_{xx} ( E_{nn}^2 + E_{ss}^2 + E_{sn}^2 + E_{ns}^2 ) \\ &\quad + b_{fx} ( E_{sf}^2 + E_{fs}^2 + E_{nf}^2 + E_{fn}^2 ) ]. \end{aligned} \end{aligned}$$

The PK2 stress may be computed through the matrix derivative with respect to the Green strain:

26 $$\begin{aligned} \varvec{S}_e = \dfrac{ \partial \varPsi }{ \partial \varvec{E} } . \end{aligned}$$

These definitions imply that the PK2 stress tensor in the fiber coordinate (*s*, *n*, *f*) representation is

27 $$\begin{aligned} \varvec{S}_e = k_e e^W \left[ \begin{matrix} b_{xx} E_{ss} &{}\ b_{xx} E_{sn} &{} \ b_{fx} E_{sf} \\ b_{xx} E_{sn} &{}\ b_{xx} E_{nn} &{} \ b_{fx} E_{nf} \\ b_{fx} E_{sf} &{}\ b_{fx} E_{nf} &{} \ b_{ff} E_{ff} \end{matrix} \right] . \end{aligned}$$

The fiber coordinate representation of the elastic stress is rotated to prolate coordinates using (23).

#### Viscous stress

The viscous stress is calculated in the prolate spheroidal coordinate system. The viscous stress may be computed by (Moulton et al. 2017)

28 $$\begin{aligned} \varvec{S}_v = k_{v} \varvec{C}^{-1} \dfrac{ \partial \varvec{C} }{\partial t} \varvec{C}^{-1}. \end{aligned}$$

The time derivatives of $\varvec{C}$ can be written in terms of derivatives of the Green strain with respect to the deformation parameters $\varvec{q}(t)$ using the chain rule:

29 $$\begin{aligned} \dfrac{ \partial \varvec{C} }{\partial t} = \sum _{i=1}^{N_q} \dfrac{\partial \varvec{C}}{\partial q_i} \dfrac{ \hbox {d}q_i }{\hbox {d}t} = 2 \sum _{i=1}^{N_q} \dfrac{\partial \varvec{E}}{\partial q_i} \dfrac{ \hbox {d}q_i }{\hbox {d}t}. \end{aligned}$$

Using this form, (28) becomes

30 $$\begin{aligned} \varvec{S}_v = 2 k_{v} \sum _{i=1}^{N_q} \left( \varvec{C}^{-1} \dfrac{ \partial \varvec{E} }{\partial q_i} \varvec{C}^{-1} \right) \frac{ \hbox {d} q_{i}}{ \hbox {d} t}. \end{aligned}$$

#### Active stress

The active contractile force of the cardiac muscle is assumed to act only in the fiber direction (Nash 1998):

31 $$\begin{aligned}{}[\varvec{S}_a]_\mathrm{fiber} = \left[ \begin{matrix} 0 &{} 0 &{} 0 \\ 0 &{} 0 &{} 0 \\ 0 &{} 0 &{} T_a \end{matrix} \right] . \end{aligned}$$

We construct a simplified model for the active tension $T_a$ using an activation function *A*(*t*), a length–tension relationship $g(\lambda _{ff})$, and a linear force–velocity relationship:

32 $$\begin{aligned} T_a = A(t) g( \lambda _{ff} ) \left( k_a + k_{av} \frac{ \partial \lambda _{ff} }{ \partial t } \right) , \end{aligned}$$

where $\lambda _{ff}$, the stretch ratio in the fiber direction, may be computed from the Green strain in the fiber direction as

33 $$\begin{aligned} \lambda _{ff} = \sqrt{2 E_{ff} + 1 }. \end{aligned}$$

The parameter $k_a$ determines the strength of the activation, while $k_{av}$ defines the relative magnitude of the force–velocity dependence. We assume a Gaussian length–tension relationship:

34 $$\begin{aligned} g(\lambda _{ff}) = \exp \left[ \dfrac{ - ( L_{s0}\lambda _{ff} - L_{s,max})^2}{ (2 L_{sw} )^2 } \right] . \end{aligned}$$

In this equation, $L_{s0}$ is an assumed sarcomere length in the unstressed myocardium, $L_{s,max}$ is the length that provides maximal force generation, and $L_{sw}$ determines the width of the Gaussian. The Gaussian shape was chosen to match the data recorded by Julian and Sollins (1975). We use a modified sine curve to approximate the time-dependence of active stress during systole:

35 $$\begin{aligned} A(t) = \left\{ \begin{array}{ll} \sin ^d(\pi t / T_a ) \quad &{} \text { during activation } \\ 0 \quad &{} \text { otherwise. } \end{array} \right. \end{aligned}$$

The time derivative in (32) may be decomposed in terms of the time-dependent deformation parameters $\varvec{q}$, and (32) gives

36 $$\begin{aligned} T_a = A(t) g(\lambda _{ff} ) \left( k_a + k_{av} \sum _{i=1}^{N_q} \dfrac{ \partial \lambda _{ff} }{\partial q_i} \frac{ \hbox {d} q_{i}}{ d t}\right) . \end{aligned}$$

The active PK2 stress in prolate coordinates is computed from (36) using the tensor rotation (23).

#### Surface tractions and chamber volume

The traction at the endocardial surface generated by LV chamber pressure is

37 $$\begin{aligned} \varvec{T} = P_\mathrm{lv} \varvec{n}, \end{aligned}$$

where $\varvec{n}$ is the surface normal in the deformed configuration and $P_\mathrm{lv}$ is the chamber pressure. The surface normals may be computed by taking the cross-product of the surface tangent vectors (supplementary material Section S8).

For the system to conserve energy, the LV chamber must be closed to define the cavity volume and work done by motion at the base must be incorporated into the virtual work equations. An appropriate closing surface is difficult to define in prolate spheroidal coordinates, and so the surface $\varGamma$ is defined in cylindrical coordinates (supplementary material Section S9) as shown in Fig. 2.

#### Virtual work differential equation system

Under the above assumptions, the virtual work equations (21) may be written as a system of ODEs, in which the kinematic variables $q_i$ and the LV pressure $P_\mathrm{lv}$ are functions of time *t*. The total PK2 stress can be expressed as

38 $$\begin{aligned} \begin{aligned} \varvec{S}&= \varvec{S}_e + \varvec{S}_a + \varvec{S}_v \\&= \varvec{S}_e + \varvec{S}_{a,0} + \sum _{j=1}^{N_q} \left( \varvec{S}_{a,q_j} \dfrac{\hbox {d} q_j}{ \hbox {d} t} + \varvec{S}_{v,q_j} \dfrac{\hbox {d} q_j}{ \hbox {d} t} \right) , \end{aligned} \end{aligned}$$

where

39 $$\begin{aligned} \begin{aligned} \varvec{S}_{a,0}&= \varvec{Q} \left[ \begin{matrix} 0 &{} 0 &{} 0 \\ 0 &{} 0 &{} 0 \\ 0 &{} 0 &{}k_a A g \end{matrix} \right] \varvec{Q}^\top \\ \varvec{S}_{a,q_j}&= \varvec{Q} \left[ \begin{matrix} 0 &{} 0 &{} 0 \\ 0 &{} 0 &{} 0 \\ 0 &{} 0 &{}k_{av} A g \dfrac{ \partial \lambda _{ff} }{\partial q_j} \end{matrix} \right] \varvec{Q}^\top \\ \varvec{S}_{v,q_j}&= 2 k_{v} \left( \varvec{C}^{-1} \dfrac{ \partial \varvec{E} }{\partial q_j} \varvec{C}^{-1} \right) . \end{aligned} \end{aligned}$$

For convenience, we introduce

40 $$\begin{aligned} \begin{aligned} \alpha _{ i,j }&= \int \limits _{\varOmega _0} \frac{ \partial \varvec{E} }{ \partial q_i } : ( \varvec{S}_{v,q_j} + \varvec{S}_{a,q_j} ) \ \hbox {d}V_0 \\ \kappa _{ i }&= \int \limits _{\varOmega _0} \frac{ \partial \varvec{E} }{ \partial q_i } : ( \varvec{S}_{e} + \varvec{S}_{a,0} )\ \hbox {d}V_0 \\ \eta _{i}&= \int _{\partial \varOmega _0} \dfrac{\partial \varvec{u}}{\partial q_i} \cdot (\varvec{v}_{\nu _0} \times \varvec{v}_{\phi _0} )\ \hbox {d}\nu _0 \hbox {d}\phi _0 \\&\quad + \int _{\varGamma } \dfrac{\partial \varvec{u}}{\partial q_i} \cdot (\varvec{v}_{l1} \times \varvec{v}_{l2} )\ \hbox {d} u_r \hbox {d}\phi _0, \end{aligned} \end{aligned}$$

where the two terms of $\eta _i$ give the work done at the endocardial surface and at the base of the LV (supplementary material Section S9.2).

With these definitions, the virtual work system (21) yields

41 $$\begin{aligned} \left\{ \begin{aligned} \alpha _{1,1} \dfrac{\hbox {d} q_1 }{ \hbox {d} t} + \cdots + \alpha _{1,N_q} \dfrac{\hbox {d} q_{N_q} }{\hbox {d} t} + \kappa _{1} - \eta _{1} P_\mathrm{lv}&= 0 \\ \alpha _{2,1} \dfrac{\hbox {d} q_1 }{\hbox {d} t} + \cdots + \alpha _{2,N_q} \dfrac{\hbox {d} q_{N_q} }{\hbox {d} t} + \kappa _{2} - \eta _{2} P_\mathrm{lv}&= 0 \\ &\ \ \vdots \\ \alpha _{N_q,1} \dfrac{\hbox {d} q_1 }{\hbox {d} t} + \cdots + \alpha _{N_q,N_q} \dfrac{\hbox {d} q_{N_q} }{\hbox {d} t} + \kappa _{N_q} - \eta _{N_q} P_\mathrm{lv}&= 0. \end{aligned} \right. \end{aligned}$$

This system has $N_q$ equations and $N_q+1$ unknowns, $\varvec{q}$ and $P_\mathrm{lv}$. An additional equation represents conservation of blood volume

42 $$\begin{aligned} \dfrac{ \partial V_\mathrm{lv} }{ \partial q_1 } \dfrac{ \hbox {d} q_1 }{ \hbox {d} t } + \cdots + \dfrac{ \partial V_\mathrm{lv} }{ \partial q_{n_q}} \dfrac{ \hbox {d} q_{n_q} }{ \hbox {d} t } - q_\mathrm{mv} + q_\mathrm{aov} = 0, \end{aligned}$$

where $q_\mathrm{mv}$ is the flow into the LV through the mitral valve and $-q_\mathrm{aov}$ represents the flow rate of the blood exiting the LV through the aortic valve. The expressions for $q_\mathrm{mv}$ and $q_\mathrm{aov}$ depend on the models chosen for the other components of the circulatory system.

Under equilibrium conditions, the time derivatives vanish and the pressure $P_\mathrm{lv}$ becomes a constant, reducing the ODE system (41) to a nonlinear algebraic system for $\varvec{q}$:

43 $$\begin{aligned} \kappa _{i} - \eta _{i} P_\mathrm{lv} = 0, \end{aligned}$$

where $i = 1, \ldots , N_q$. The solution to this nonlinear system may be approximated by a nonlinear solver such as Newton’s method.

#### Numerical solution of the virtual work system

The system (41,42) is an initial value problem for $\varvec{q}$ and $P_\mathrm{lv}$. We solve this system by setting initial values of $\varvec{q}_0$, determining the pressure $P_\mathrm{lv}$ in that state, and integrating in time using an explicit scheme such as a Runge–Kutta (RK) method. Time is discretized as $t = m \varDelta t$. When solving for the $m+1$ time step, the values of the coefficients $\varvec{\alpha }$, $\varvec{\kappa }$, and $\varvec{\eta }$ are computed at time step *m*. The reference myocardial domain $\varOmega _0$ is discretized into a $N_\mu \times N_\nu \times N_\phi$ grid to facilitate the numerical evaluation of the coefficients (40). The volume integrals are approximated using a Simpson’s rule that integrates through $\mu _0$, $\nu _0$, and $\phi _0$, in that order (supplementary material Section S11).

### Dynamic model solution verification

To validate the proposed approach, we describe two sets of simulations. In the first, we validate the model by comparison to analytic equilibrium solutions to the strong form equations (Malvern 1969):

44 $$\begin{aligned} \begin{aligned}&\text {div } \varvec{\sigma }+ \varvec{b} = \varvec{0} \\&\varvec{t} = \varvec{\sigma }\varvec{n} \text { on } \partial \varOmega , \end{aligned} \end{aligned}$$

and demonstrate the ability of the proposed approach to accurately reproduce these solutions. In the second part, we show the ability of the characteristic deformation model to produce realistic simulations of the cardiac cycle.

#### Comparisons using the strong form of the equilibrium equations

We verify the variational method solutions using two types of analytic solutions. In the supplementary material Section S12, we evaluate the ability of the model to solve for the deformation of an infinitely extended hollow cylinder. Here, we test the ability of the variational method described in Sect. 2.3 to recover an exact solution derived from the strong form of the equilibrium equations (44). In the absence of viscous and active stress terms, the strong form of the equilibrium equations requires

45 $$\begin{aligned} \text {div } \varvec{\sigma }_{e} + \varvec{b} = 0. \end{aligned}$$

Typically, the body force $\varvec{b}$ represents gravity. Here, however, we set

46 $$\begin{aligned} \varvec{b} = \varvec{b}_f = - \text {div } \varvec{\sigma }_{ef}, \end{aligned}$$

where $\varvec{\sigma }_{ef} = \varvec{\sigma }_{e}( \varvec{q}_f )$ is the elastic stress associated with a chosen set of kinematic variables $\varvec{q} = \varvec{q}_f$ (supplementary material Section S13). Also, we set the surface tractions to correspond to the chosen elastic stress field

47 $$\begin{aligned} \varvec{t} = \varvec{t}_f = \varvec{\sigma }_{ef} \varvec{n}_f, \end{aligned}$$

where $\varvec{n}_f$ is the outward surface normal in the deformed configuration at the desired solution state $\varvec{q}_f$. The modified form of the virtual work system (21) including the body force $\varvec{b}_f$ and the traction $\varvec{t}_f$ is

48 $$\begin{aligned} \int _{\varOmega _0} \varvec{S}: \dfrac{\partial \varvec{E} }{ \partial q_i } \hbox {d}V - \int _{ \varOmega } \varvec{b}_f \cdot \dfrac{\partial \varvec{u} }{ \partial q_i } \hbox {d}v - \int _{\partial \varOmega } \varvec{t}_f \cdot \dfrac{\partial \varvec{u} }{ \partial q_i } \hbox {d}s = 0, \end{aligned}$$

where the traction $\varvec{t}_f$ acts over the full myocardial boundary. Under these assumptions, the solution to (48) should recover the chosen kinematic variables $\varvec{q}_f$. The solution is obtained using the Fourier-series deformation model described in Sect. 2.2.2 with a set of $N_q = 37$ deformation modes. The resulting equilibrium solutions are compared to the original assumed solution.

#### Cardiac cycle simulations

To illustrate the degree of kinematic freedom that is required for describing cardiac dynamics, we simulate the cardiac cycle in two cases. First, we simulate the full cardiac cycle. Second, we examine the fiber stretch and stress distributions under end-systolic and end-diastolic equilibrium conditions. In both sets of simulations, we compare models that use the Fourier-series deformation modes (11) with 8, 23, and 46 DOF.

**Fig. 4:**
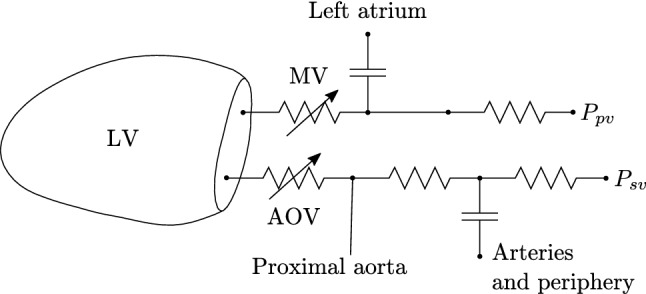
Simplified preload and afterload model using variable resistance valves. The pressures in the pulmonary veins $P_\mathrm{pv}$ and systemic veins $P_\mathrm{sv}$ are assumed to be constant for simplicity

We use simplified lumped-parameter models (Moulton et al. 2017) (supplementary material Section S10) to describe the preload and afterload systems, as illustrated in Fig. 4. The aortic valve (AOV) and mitral valve (MV) are modeled by variable resistances that have a nonlinear dependence on the pressure variable $P_\mathrm{lv}$ (S67,S66). The full system (41,42) therefore requires a nonlinear solver to evaluate the derivative terms necessary for the time integrator. We use Newton’s method to evaluate the time derivatives (supplementary material Section S11).

## Results

### Evaluation of the kinematic model using cardiac MRI

**Fig. 5:**
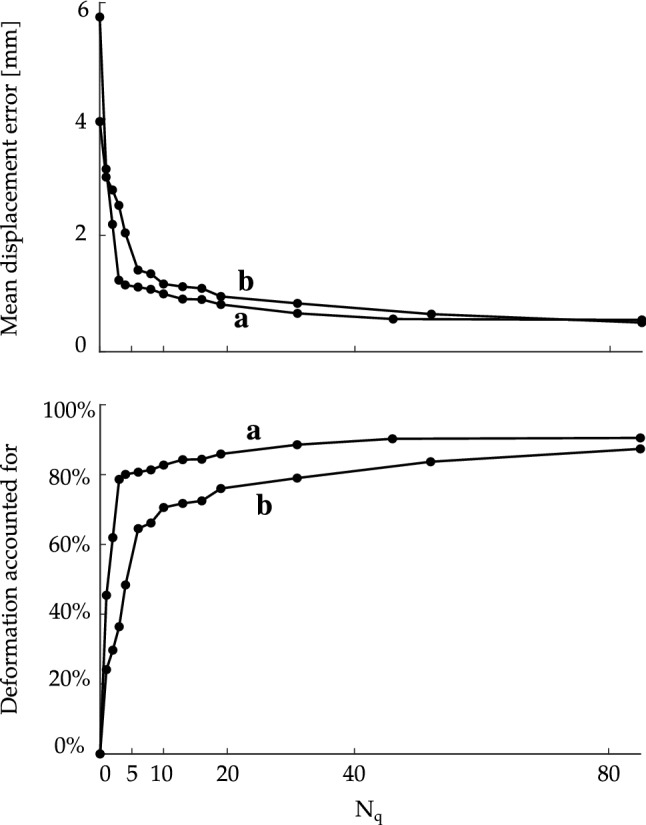
Top: mean displacement error between registered MRI displacements and those produced by the optimized kinematic model with an increasing number of modes $N_q$ calculated using (49). Bottom: deformation accounted for with $N_q$ deformation modes calculated using (50). Curve **a** used data from a healthy volunteer, while curve **b** used data from a patient with dilated cardiomyopathy

As described in Sect. 2.2.4, we evaluated the ability of the kinematic modes (see Sect. 2.2.2) to describe LV motion measured from tagged cardiac MRI. We show the result of that analysis in Fig. 5.

We found that 6 deformation modes were sufficient to reduce the mean displacement error from a maximum of 5.8 mm to 1.1 mm in the normal case and from 4.0 mm to 1.4 mm in the pathological case. We computed the mean displacement error as the volume-averaged error

49 $$\begin{aligned} E(\varvec{q}; N) = \dfrac{1}{V_\mathrm{m}} \int _{\varOmega _0} | \varvec{u}_\mathrm{m}( \varvec{x}_0; \varvec{q} ) - \varvec{u}_{r}(\varvec{x}_0) | \ \hbox {d}V, \end{aligned}$$

where $|\cdot |$ is the Euclidean norm, $V_\mathrm{m}$ is the myocardial wall volume, $\varvec{u}_\mathrm{m}$ is the Cartesian representation of the displacement according to the selected *N* deformation modes $\varvec{q}$, and $\varvec{u}_r$ is the displacement data registered from the cardiac MRI. Thus, 6 deformation modes were capable of accounting for 81% of the deformation in the healthy case and 64% in the case of dilated cardiomyopathy. When the number of deformation modes was increased to 19, these percentages increased to 86% and 76%, respectively. We computed the deformation accounted for as the mean error reduction

50 $$\begin{aligned} \dfrac{ E(\varvec{q};0) - E(\varvec{q};N) }{ E(\varvec{q};0) } \cdot 100\%. \end{aligned}$$

The percentages of deformation accounted for (averaged across both cases) by the first 4 displacement modes were 35%, 11%, 12%, and 7%. The first three modes (that account for over 50% of the deformation across these two examples) were, respectively, uniform expansion, uniform twist, and uniform elongation. Diminishing returns were observed after 10 deformation modes as each additional deformation mode yielded less than a 1% error reduction.

### Comparisons using the strong form of the equilibrium equations

**Fig. 6:**
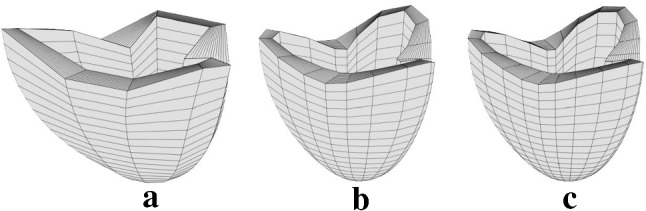
Deformed model solutions to the forced virtual work system (48) used for validating the numerical solutions. **a** Model solution with $N_\phi = 10$ integration points. With only ten integration points in the $\phi$ direction, the model has not yet converged to the analytic solution (**c**). **b** Model solution with $N_\phi = 20$, showing convergence to the expected solution (**c**). **c** Analytic solution generated using the forcing term $\varvec{b}$ in the strong form equations (44)

As shown in the supplementary material Figure S5, the proposed variational approach accurately predicts the deformation of the hollow cylinder. Here, we test solutions to the virtual work system (43) using the body and traction forcing terms described in Sect. 2.4.1. The expected solution has $\varvec{q}_f = \varvec{0}$ except $q_1 = q_2 = q_{15} = q_{30} = 0.1$ and $q_{16} = -0.1$. These parameters were chosen to provide significant asymmetric variation in all three coordinates ($\mu$, $\nu$, and $\phi$). The expected solution is graphed along with the model solutions in Fig. 6. The computation is repeated at several levels of resolution in the numerical integration grid in the $\phi _0$ direction to illustrate numerical convergence to the expected solution. The displacement error is defined as the average distance between the positions at the expected solution $\varvec{q}_f$ and the model solution $\varvec{q}$:

51 $$\begin{aligned} e_{\varvec{x}} = \dfrac{1}{N_\mathrm{nodes}} \sum _{i=1}^{N_\mathrm{nodes}} |\varvec{x}_i(\varvec{q}) - \varvec{x}_i(\varvec{q}_f) |. \end{aligned}$$

The kinematic variable error is defined as the mean absolute difference between the computed and the expected solution:

52 $$\begin{aligned} e_{\varvec{q}} = \dfrac{1}{N_{q}} \sum _{i=1}^{N_{q}} | q_i - q_{fi} |. \end{aligned}$$

**Fig. 7:**
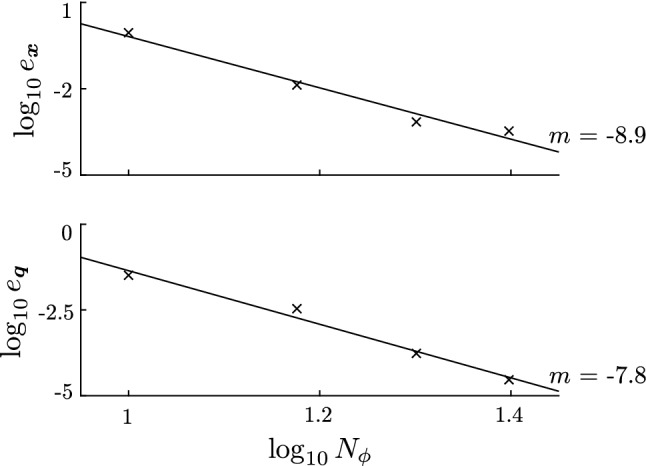
Log–log plots of the deviations between model and exact solutions with increasing resolution of the $\phi$ integration grid ($\times$). Upper plot: Average displacement deviation. Lower plot: Mean absolute deviations of kinematic variables. The slopes of the plotted linear regression lines indicate the convergence order for each case

The convergence of these variables is shown in Fig. 7.

**Fig. 8:**
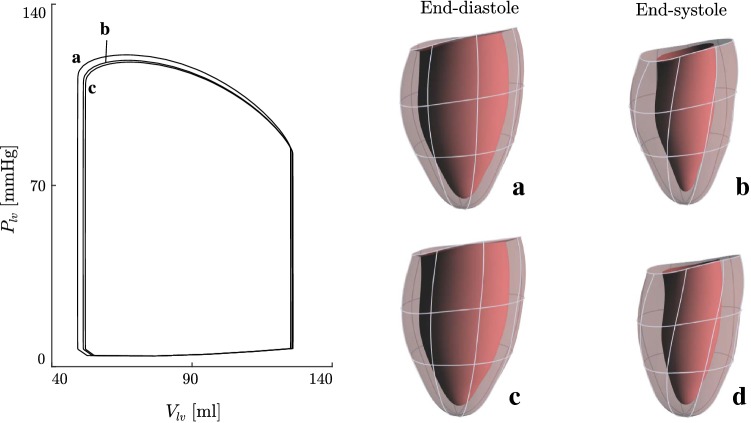
Simulations of normal cardiac function under variations in the number of kinematic variables $\varvec{q}$. The deformation model described in Sect. 2.2.2 is used with **a**$N_q = 8$ , **b**$N_q = 23$, and **c**$N_q = 46$ deformation parameters. Lines plotted together with the LV shapes illustrate LV deformations and do not indicate the resolution of the numerical integration grid

### Cardiac cycle simulations

Figure 8 illustrates simulations of the cardiac cycle. The values of material parameters of the LV and the parameters of the lumped circulatory system were chosen to reproduce normal cardiac function (supplementary material Tables S1 and S2). The three simulations illustrate changes to cardiac function and deformation as the number of kinematic parameters is increased.

**Fig. 9:**
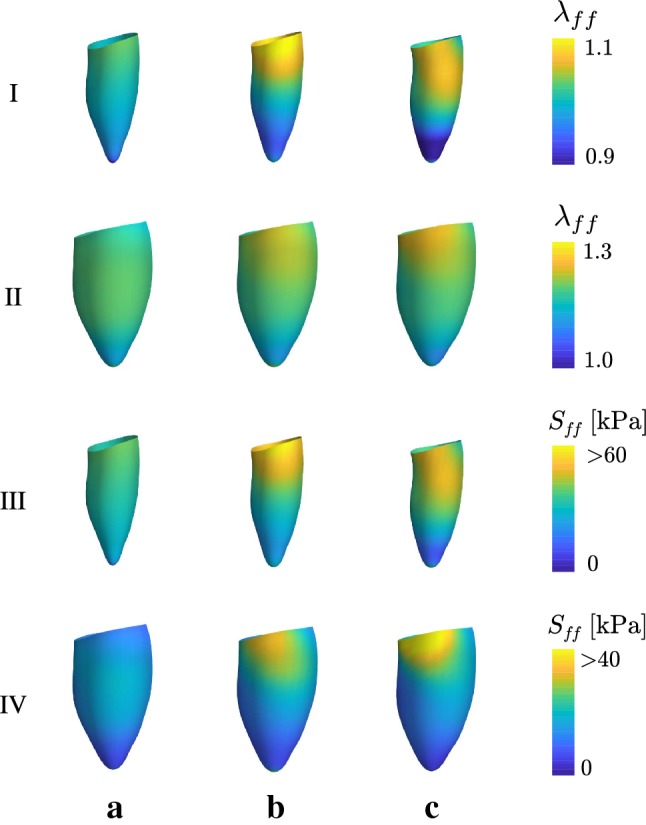
Distributions of stretch ratios $\lambda _{ff}$ and deviatoric PK2 stress ($S_{ff}$) in the fiber direction at end-systole (I, III) and end-diastole (II, IV). Values are recorded at the endocardial wall. The deformation model described in Sect. 2.2.2 is used with **a**$N_q = 8$ , **b**$N_q = 23$, and **c**$N_q = 46$ deformation parameters

Figure 9 shows fiber stretch ratios $\lambda _{ff}$ at end-systole and end-diastole computed using (33) as well as the PK2 stress in the fiber direction $S_{ff}$. The surface plots illustrate local variations in the kinematic and dynamic quantities at the endocardial wall. The three simulations demonstrate changes to the fiber stretch and stress distributions as the number of kinematic variables is increased.

## Discussion

The development of theoretical models for LV mechanics has been an active area of investigation for decades. A variety of approaches have been used, ranging from varying elastance models, in which the geometry of the LV is not explicitly represented, to finite element models based on detailed descriptions of LV geometry. While this work has provided important insights into cardiac mechanics, all such models have limitations in terms of their applicability. The simpler models have limited ability to make use of information about LV shape and motion, while the more complex models involve challenging issues of parameter specification and impose heavy computational requirements. The goal of the present work is to develop approaches for modeling LV mechanics that can incorporate information about LV shape and deformation, as derived, for example, from echocardiography, and yet are computationally efficient so that they can be used to simulate the cardiac cycle with computational times that would be compatible with clinical applications.

### Kinematics

In previous work, we developed models for LV mechanics based on a cylindrical model (Moulton and Secomb 2013) and an axisymmetric spheroidal model (Moulton et al. 2017). In both cases, three deformation modes (radial and axial contraction, and torsion) were used to represent the dominant modes of LV deformation during the cardiac cycle. An important feature of these models was that the assumed deformation modes inherently conserve myocardial volume. Therefore, the resulting systems of equations do not involve stiff constraints resulting from the very high bulk modulus of cardiac tissue, allowing more efficient numerical solution. However, both of these models were restricted to geometries with rotational symmetry about a central axis. In the present model, this constraint is relaxed, and non-axisymmetric reference LV shapes and non-axisymmetric modes of deformation are simulated. The deformation modes retain the property of conserving myocardial volume. The number of deformation modes is increased relative to the earlier models, with an arbitrary number of Fourier-series modes.

We evaluated this kinematic model using two tagged MRI data sets. The results are graphed in Fig. 5. We found that, in a normal volunteer, 5 modes were sufficient to capture 80% of the deformation from end-systole to end-diastole. In a patient with dilated cardiomyopathy, 10 modes were sufficient to capture 70% of the deformation. This illustrates that the majority of LV deformation was accounted for with relatively few deformation modes. This analysis suggests that the characteristic deformation model could be effectively employed to study cardiac function in both normal and pathological cases.

The notion of understanding the heart in terms of a limited number of modes has previously been developed using the Cardiac Atlas Project database, which includes more than 3000 cardiac MRI studies (Fonseca et al. 2011). This database has largely been analyzed using principal component analysis (PCA) of LV shapes at end-systole and end-diastole (Zhang et al. 2014). Farrar et al. (2016) demonstrated that the first 5 modes of the PCA were able to account for 58% of the end-systolic to end-diastolic motion in asymptomatic populations. Their result that 5 modes are sufficient to recapitulate much of the cardiac motion agrees well with our analysis. In this work, we developed deformation modes using a first principles approach by constructing a geometry and deformation modes in prolate coordinates that are naturally suited to the LV shape. While these modes are sufficient to recapitulate much of the cardiac deformation in two tagged MRI data sets, kinematic modes with improved physical relevance could be developed through a statistical analysis of cardiac motion across a large imaging database (such as the PCA used by Farrar et al. 2016).

### Dynamics

In order to validate the dynamic model and its numerical solution, we applied three tests. First we compared the model solution to the analytic solution for an incompressible cylinder of a Mooney–Rivlin material under expansion. Figure S5 shows that the model accurately predicts the deviatoric stresses with only five integration points in the $\mu$ direction. Isotropic stresses contribute no work in incompressible deformations, and are therefore not computed within the model framework. However, the isotropic stress may be computed from the model solution. Figure S5 shows that the isotropic stress computed in the model solution agrees well with the analytic result. Secondly, we constructed an exact solution to the strong form equations using asymmetric deformation modes, and demonstrated that the variational approach converged to the strong form solution as the numerical integration grid was resolved (Fig. 7). Because the strong form solution is independent of the virtual work formulation, these simulations verify the virtual work system (41), as well as the numerical implementation.

The third type of test involved simulations of normal cardiac function (Fig. 8). We computed three model solutions with varying degrees of kinematic freedom. Simulation **a** had $N_q = 8$ degrees of freedom and required 2.3 seconds per cardiac cycle to compute in parallel on an Intel i9-7980XE workstation with 18 cores clocked at 2.6 GHz. Simulation **b** had $N_q = 23$ degrees of freedom and required 9.1 s/cycle, while simulation **c** had $N_q = 46$ and required 27 s/cycle. By comparison, (Kerckhoffs et al. 2007) reported that individual time steps with an FEM model required 2 min to compute. This implies that each cardiac cycle computed with 400 time steps (the temporal resolution used here) would require 13 h. While recent advances in parallel computing would likely improve that estimate, the method presented here still presents an efficient alternative, especially in the cases where 8 or 23 deformation modes are used.

Despite the variation of the degrees of freedom and consequent computation time increases, only minor differences are visible in the cardiac cycle PV-loop between simulations (Fig. 8). End-diastolic and end-systolic shapes are similar in terms of aggregated parameters: volume, long-axis length, and short-axis radius. The work done with 8 deformation modes (1.084 J/cycle) differs from the work done with 46 deformation modes (1.028 J/cycle) by $5.1\%$. In addition, the volume, long-axis length, and short-axis radius at end-diastole differ by $0.7\%$, $0.6\%$, and $2.6\%$, respectively. These simulations support the use of a restricted set of deformation modes to represent LV dynamics, since the inclusion of additional modes had only small effects on these parameters. This result suggests that, for studies based on aggregated parameters or limited data, the model with 8 modes would be adequate.

Figure 9 shows the effects of varying the number of deformation modes on distributions of stress and strain. Rows (I) and (II) show that all three models have similar mean stretch ratios in the fiber direction. However, as the number of kinematic variables is increased, more localized spatial variations are developed with greater magnitude. These variations result from the nonsymmetric initial shape of the LV. The PK2 fiber stress distributions (III, IV) show similar trends. The stress at end-systole is almost linearly correlated with the fiber stretch $\lambda _{ff}$ due to the stretch dependence of the active stress generation described in Eq. (32).

### Limitations and future development

In the approach presented here, some restrictions are imposed on the allowable deformation modes. The functions $\phi (\nu _0, \phi _0)$ and $\nu ( \nu _0, \phi _0 )$ mapping the reference to the deformed configuration are assumed to be independent of $\mu _0$. This assumption makes possible the integration of (7) with respect to $\mu _0$, giving an implicit algebraic equation (9) for $\mu (\mu _0,\nu _0, \phi _0)$. At the same time, this assumption largely restricts shearing motions within the myocardium to the torsional component. The approach presented here could be extended to allow the other two components of shear deformation. In that case, $\mu$, $\phi$ and $\nu$ would all be functions of $\mu _0$, $\nu _0$ and $\phi _0$, and the $\mu$ mapping would not be expressible in algebraic form.

While providing a natural framework for describing the LV geometry and kinematics, the prolate spheroidal coordinate system suffers from limits to the deformable freedom allowed through the apex due to the singularities at the axis. While the incompressible kinematic model described in Sect. 2.2.1 is dependent on this choice, the variational formulation used to compute LV dynamics in terms of a limited number of kinematic variables is not. It would be possible to describe a geometry and analogous kinematic model in any curvilinear coordinate system, such as spherical or Cartesian coordinates, and the formulation of the dynamic model presented in Sect. 2.3 would apply.

As shown in Fig. 5, a relatively small number of modes accounts for a large proportion of the deformation, as defined in terms of displacement error. The stress and strain depend on derivatives of the displacement field and, consequently, are more strongly affected by inclusion of higher-order displacement modes. A substantially larger number of modes would be required to achieve numerical convergence of the stress and strain fields. The present model is not well suited for computing stress and strain at high spatial resolution, for which a FEM approach would be more suitable.

While we have only described the mechanics of the LV, the framework developed here is naturally extendable to a bi-ventricular geometry. The right ventricle can be directly added to the variational equations (15). Displacement modes can be constructed separately for the RV, although motion continuity would be required at the LV-RV boundary. Further, with appropriate simplified models for the heart valves, this approach could be extended to produce efficient simulations of a four-chamber heart model. The kinematics of each additional chamber would primarily be described by deformation modes that extend only over that region, implying that the increase in computational cost would be generally additive.

A simplified description of myocardial activation and force generation is used. The model for active force takes into account the length–tension and force–velocity characteristics of cardiac muscle. The Frank–Starling effect, in which force generation depends on end-diastolic fiber strain, is not included, although this effect can be represented by a simple modification to (32) (Moulton et al. 2017). The time-dependence of force generation during systole is represented by a spatially independent activation function $A = A(t)$, and the effects of the time-dependent spatial spread of activation are not included here. Such effects could be introduced in the present approach by using a spatially varying activation function $A = A(\varvec{x},t)$, provided that the deformation modes were chosen to accommodate the resulting cardiac motions.

### Conclusion

A method for simulating LV dynamics using a limited number of deformation modes, which was originally developed for axisymmetric geometries (Moulton et al. 2017), is here extended to general three-dimensional LV shapes and deformations. The method is computationally efficient: simulation of one cardiac cycle using the 23 deformation mode model takes approximately 9 s on a personal computer. Addition of modes beyond this number has only slight effects on overall parameters describing LV function, although local distributions of stress and strain are still affected. The method is suitable for a range of applications in which FEM simulations at high spatial resolution would be computationally impractical. Such applications include systemic exploration of effects of changing parameters describing cardiac mechanics, estimation of such parameters from echocardiographic imaging data, simulations performed over multiple cardiac cycles, and estimation of spatially dependent fiber stresses and strains for use in models of cardiac remodeling.

## Electronic supplementary material

Below is the link to the electronic supplementary material.

Supplementary material 1 (pdf 790 KB)
