# Supplementary material for: Modeling left ventricular dynamics with characteristic deformation modes
Source: Biomech Model Mechanobiol. 2019 May 25;18(6):1683–96. doi: 10.1007/s10237-019-01168-8 (PMC6825036; doi:10.1007/s10237-019-01168-8)
Supplement: Supplementary file 1 — Supplementary material 1 (pdf 790 KB) [file 10237_2019_1168_MOESM1_ESM.pdf]

Modeling left ventricular dynamics with characteristic deformation modes

Supplementary material

Brian D. Hong · **Michael J. Moulton** · **Timothy W. Secomb**

S1 Model parameters

In Tables S1 and S2 we outline the parameters used for all simulations of regular cardiac function, unless otherwise noted in the text.

Table S1 LV model parameters.

Parameter	Description	Value
k_e	Stiffness multiplier	2.0 kPa
b_{ff}	Fiber direction stiffness coeff.	3.5
b_{xx}	Transverse direction stiffness coeff.	2.0
b_{fx}	Shearing stiffness coeff.	1.0
k_v	Viscous stress coeff.	0.1 kPa·s
k_a	Active stress coeff.	160 kPa
k_{av}	Active stress force-velocity coeff.	10 kPa·s
L_{s0}	Sarcomere slack length	1.82 μm
$L_{s,max}$	Sarcomere length with maximal force	2.4 μm
L_{sw}	Additional length-tension parameter	0.435 μm
$\psi_{in,b0}$	Endocardial equatorial fiber angle	60°
$\psi_{out,b0}$	Epicardial equatorial fiber angle	-60°
T_c	Cardiac cycle period	1 s
T_a	Active contraction duration	0.5 s
N_μ	Integration points in the μ direction	7
N_ν	Integration points in the ν direction	11
N_ϕ	Integration points in the ϕ direction	15
Δt	Time step for dynamic simulation	0.025 s

Table S2 Lumped system parameters.

Parameter	Description	Value
$R_{mv,o}$	Open mitral valve flow resistance	0.001 kPa·s/ml
$R_{mv,c}$	Closed mitral valve flow resistance	10 kPa·s/ml
$R_{mv,o}$	Open aortic valve flow resistance	0.001 kPa·s/ml
$R_{mv,c}$	Closed aortic valve flow resistance	10 kPa·s/ml
R_{pv}	Pulmonary vein flow resistance	0.001 kPa·s/ml
R_{pao}	Proximal aortic flow resistance	0.0005 kPa·s/ml
R_{sa}	Systemic arterial flow resistance	0.008 kPa·s/ml
R_{sp}	Systemic peripheral flow resistance	0.18 kPa·s/ml
C_{la}	Left atrial compliance	60 ml/kPa
C_{sa}	Arterial compliance	6 ml/kPa
C_{sp}	Systemic peripheral compliance	10 ml/kPa
P_{pv}	Fixed pulmonary vein pressure	1.2 kPa
P_{sv}	Fixed systemic vein pressure	1.2 kPa

S2 Prolate spheroidal bicubic splines

In this section we define bicubic splines in the prolate spheroidal coordinate system, providing a general framework for constructing functions in these coordinates. We define the reference endocardial and epicardial surfaces using these splines, illustrated in Figure S1. Although not shown here, these functions may also be used to construct arbitrary deformation modes within the general kinematic framework. While we have termed our construction "bicubic splines," we do not actually compute spline conditions. Rather, we construct a function from local bicubics which have continuous values, first derivatives, and first cross derivatives at the boundaries.

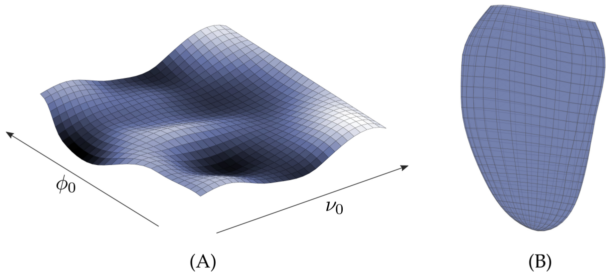

Fig. S1 Example of a reference endocardial surface (B) generated by a bicubic spline function μ_{in0} graphed in (A).

We define bicubic functions on the $\nu \times \phi$ plane on the domain

$$\phi \in [0, 2\pi) \text{ and } \nu \in [\nu_{min}, \pi]. \tag{S1}$$

Bicubic splines are constructed by partitioning the domain into N_ν sections in the ν direction and N_ϕ sections in the ϕ

direction. We denote the full bicubic spline function $f(\nu,\phi)$ which is composed of $N_\nu \cdot N_\phi$ local bicubic functions $f_{i,j}(\nu,\phi)$ indexed by $i = 1,\ldots,N_\nu$ and $j = 1,\ldots,N_\phi$. There are a total of $N_\nu + 1$ nodes in the ν direction (although the last node is degenerate) and N_ϕ nodes in the ϕ direction (due to the periodicity). The local function domains are illustrated in Figure S2.

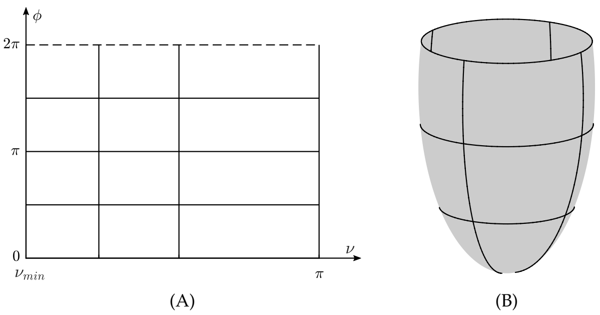

Fig. S2 Spline domain with $N_\nu = 3$ and $N_\phi = 4$. (A) shows the spline domains in their native coordinates while (B) depicts the same spline domains on a constant μ surface in their Cartesian representation.

We construct the local bicubic functions on rescaled coordinates $u_\nu, u_\phi \in [0,1]$ defined by

$$u_\nu = \frac{\nu}{\Delta\nu_i} \text{ and } u_\phi = \frac{\phi}{\Delta\phi}, \tag{S2}$$

where $\Delta\nu_i$ and $\Delta\phi$ are the widths of each local spline domain. The ϕ spacing is fixed to

$$\Delta\phi = \frac{2\pi}{N_\phi}. \tag{S3}$$

The ν spacing is variable to allow for more consistent spacing in Cartesian coordinates, as illustrated in Figure S2. The bicubic functions are defined locally as

$$f_{i,j}(u_\nu,u_\phi) = \sum_{m,n=0}^{3} p_{m,n} u_\nu^m u_\phi^n. \tag{S4}$$

The full spline function $f(\nu,\phi)$ is evaluated by identifying the correct region of the domain and then computing f from (S4).

The nodes at the apex $\nu = \pi$ are degenerate. We can directly specify the value of f at these nodes, but not the derivatives. It is possible, however, to define nonaxisymmetric derivative variations in the spline function at $\nu = \pi$ while preserving continuity of the derivatives through the apex. We define a parameter vector $\boldsymbol{b} = [b_0, b_1, b_2]$ by

$$b_0 = f|_{apex} \qquad b_1 = \left.\frac{\partial f}{\partial \tilde{x}}\right|_{apex} \qquad b_2 = \left.\frac{\partial f}{\partial \tilde{y}}\right|_{apex} \tag{S5}$$

where $\tilde{x}$ and $\tilde{y}$ are

$$\tilde{x} = \sin\nu\cos\phi \qquad \tilde{y} = \sin\nu\sin\phi. \tag{S6}$$

Writing these conditions in terms of prolate-spheroidal coordinates and evaluating at the apex implies

$$\left.\frac{\partial f}{\partial \phi}\right|_{\nu=\pi} = 0 \qquad \text{and} \qquad \left.\frac{\partial f}{\partial \nu}\right|_{\nu=\pi} = b_1\cos\phi + b_2\sin\phi. \tag{S7}$$

To maintain continuity of the derivatives through the apex the local bicubics must also satisfy

$$\left.\frac{\partial f^2}{\partial\phi\partial\nu}\right|_{\nu=\pi} = -b_1\sin\phi + b_2\cos\phi. \tag{S8}$$

The free values that determine the shape of the prolate spline function f are the function values, derivatives, and first cross derivative at the nodes between spline regions. In other words, at each node we assign

$$f,\ \frac{\partial f}{\partial \nu},\ \frac{\partial f}{\partial \phi},\ \text{and } \frac{\partial^2 f}{\partial\nu\partial\phi}.$$

We define the full set of free parameters

$$\boldsymbol{s} = \left[\boldsymbol{b}, \boldsymbol{f}, \frac{\partial \boldsymbol{f}}{\partial\nu}, \frac{\partial \boldsymbol{f}}{\partial\phi}, \frac{\partial^2 \boldsymbol{f}}{\partial\nu\partial\phi}\right]. \tag{S9}$$

This implies a total of $N_s = 3 + 4N_\nu N_\phi$ free parameters that define f. The values of the function and derivatives at the degenerate nodes are fully determined by the first three values of $\boldsymbol{s}$ together with the conditions (S7) and (S8).

The local bicubic functions $f_{i,j}$ defined in (S4) have 16 coefficients $p_{m,n}$. Each local function is bordered by 4 nodes where the values of

$$f,\ \frac{\partial f}{\partial \nu},\ \frac{\partial f}{\partial \phi},\ \text{and } \frac{\partial^2 f}{\partial\nu\partial\phi}$$

are assigned by the input parameter vector $\mathbf{s}$. The 16 coefficients $p_{m,n}$ are exactly determined by the 16 conditions at each node and may be computed by inverting the matrix that prescribes these conditions.

S3 Incompressibility condition solution

Equation (9) is completed with f_c, the values of which are determined by displacements of the endocardial wall μ_{in} through

$$f_c = a^3\left[\cosh\mu_{in0}\left(\frac{1}{3}\cosh^2\mu_{in0} - \cos^2\nu_0\right) - \frac{1}{3} + \cos^2\nu_0\right] - a^3 R\left[\cosh\mu_{in}\left(\frac{1}{3}\cosh^2\mu_{in} - \cos^2\nu\right) - \frac{1}{3} + \cos^2\nu\right]. \tag{S10}$$

The mapping equation (9) is an implicit cubic equation of $\cosh\mu$. We write the solution in terms of convenience variables that provide a computationally efficient code implementation:

$$\begin{aligned} s_0 &= a^3\left[\cosh\mu_0\left(\frac{1}{3}\cosh^2\mu_0-\cos^2\nu_0\right)-\left(\frac{1}{3}-\cos^2\nu_0\right)\right] \\ s_1 &= -\cos^2\nu \\ s_2 &= -\frac{s_0-f(\nu_0,\phi_0)}{a^3K(\nu_0,\phi_0)\left(\dfrac{\sin\nu}{\sin\nu_0}\right)}-\left(\frac{1}{3}+s_1\right). \end{aligned} \tag{S11}$$

The mapping equation (9) is equivalent to

$$u^3+3s_1u+3s_2=0,\ \text{where } u=\cosh\mu, \tag{S12}$$

which has the solution

$$u=\frac{\sqrt[3]{\sqrt{4s_1{}^3+9s_2{}^2}-3s_2}}{\sqrt[3]{2}}-\frac{s_1\sqrt[3]{2}}{\sqrt[3]{\sqrt{4s_1{}^3+9s_2{}^2}-3s_2}}. \tag{S13}$$

The solution to the mapping equation is therefore

$$\mu=\cosh^{-1}u. \tag{S14}$$

S4 Apex cut-off function

We define a simple C^1 function for sending functions to zero near the apex:

$$G(\nu_0)=\begin{cases}\dfrac{1}{2}\left[1+\cos\left(\pi\dfrac{\nu_0-\nu_T}{\nu_T-\pi}\right)\right]; & \nu_0\geq\nu_T \\ 1; & \nu_T<\nu_0\end{cases}, \tag{S15}$$

where ν_T is a fixed transition point near the apex.

S5 Deformation function derivatives

Computation of the deformation gradient tensor (5) requires derivatives of the displacement functions. Here, we give the analytic form of these derivatives using convenience variables to simplify the expressions. We define convenience variables

$$A=\frac{1}{3}\cosh^3\mu-\cosh\mu\cos^2\nu-\frac{1}{3}+\cos^2\nu \tag{S16}$$

$$B=\frac{1}{3}\cosh^3\mu_0-\cosh\mu_0\cos^2\nu_0-\frac{1}{3}+\cos^2\nu_0 \tag{S17}$$

$$-\frac{1}{a^3}f_c(\nu_0,\phi_0) \tag{S18}$$

$$K=\frac{\partial\nu}{\partial\nu_0}\frac{\partial\phi}{\partial\phi_0}-\frac{\partial\nu}{\partial\phi_0}\frac{\partial\phi}{\partial\nu_0} \tag{S19}$$

The partial derivatives of these functions are

$$B_{\nu_0}=2\cos\nu_0\sin\nu_0(\cosh\mu_0-1)-\frac{1}{a^3}\frac{\partial f_c}{\partial\nu_0} \tag{S20}$$

$$B_{\phi_0}=-\frac{1}{a^3}\frac{\partial f_c}{\partial\phi_0} \tag{S21}$$

$$R_{\nu_0}=\frac{\sin\nu_0\cos\nu\dfrac{\partial\nu}{\partial\nu_0}-\sin\nu\cos\nu_0}{\sin^2\nu_0}K+\frac{\sin\nu}{\sin\nu_0}K_{\nu_0} \tag{S22}$$

$$R_{\phi_0}=\frac{\cos\nu}{\sin\nu_0}\frac{\partial\nu}{\partial\phi_0}K+\frac{\sin\nu}{\sin\nu_0}K_{\phi_0} \tag{S23}$$

$$K_{\nu_0}=\frac{\partial^2\nu}{\partial\nu_0^2}\frac{\partial\phi}{\partial\phi_0}+\frac{\partial\nu}{\partial\nu_0}\frac{\partial^2\phi}{\partial\phi_0\partial\nu_0}-\frac{\partial^2\nu}{\partial\phi_0\partial\nu_0}\frac{\partial\phi}{\partial\nu_0}-\frac{\partial\nu}{\partial\phi_0}\frac{\partial^2\phi}{\partial\nu_0^2} \tag{S24}$$

$$K_{\phi_0}=\frac{\partial^2\nu}{\partial\nu_0\phi_0}\frac{\partial\phi}{\partial\phi_0}+\frac{\partial\nu}{\partial\nu_0}\frac{\partial^2\phi}{\partial\phi_0^2}-\frac{\partial^2\nu}{\partial\phi_0^2}\frac{\partial\phi}{\partial\nu_0}-\frac{\partial\nu}{\partial\phi_0}\frac{\partial^2\phi}{\partial\nu_0\partial\phi_0}. \tag{S25}$$

The partial derivatives of the mapping equation (16) yield the derivatives of μ:

$$\frac{\partial\mu}{\partial\mu_0}=\frac{\sinh\mu_0\sin\nu_0(\cosh^2\mu_0-\cos^2\nu_0)}{K\sinh\mu\sin\nu(\cosh^2\mu-\cos^2\nu)} \tag{S26}$$

$$\frac{\partial\mu}{\partial\nu_0}=\frac{B_{\nu_0}+2R\cos\nu\sin\nu\dfrac{\partial\nu}{\partial\nu_0}(1-\cosh\mu)-R_{\nu_0}A}{R\sinh\mu(\cosh^2\mu-\cos^2\nu)} \tag{S27}$$

$$\frac{\partial\mu}{\partial\phi_0}=\frac{B_{\phi_0}+2R\cos\nu\sin\nu\dfrac{\partial\nu}{\partial\phi_0}(1-\cosh\mu)-R_{\phi_0}A}{R\sinh\mu(\cosh^2\mu-\cos^2\nu)}. \tag{S28}$$

S6 Tagged MRI data

We used tagged cardiac MRI data (illustrated in Figure S3) to evaluate the kinematic model. These data were published previously by Kar et al. (2014) and are used here with permission. As noted in that work, subjects signed informed consents in accordance with the university's Institutional Review Board (IRB) guidelines.

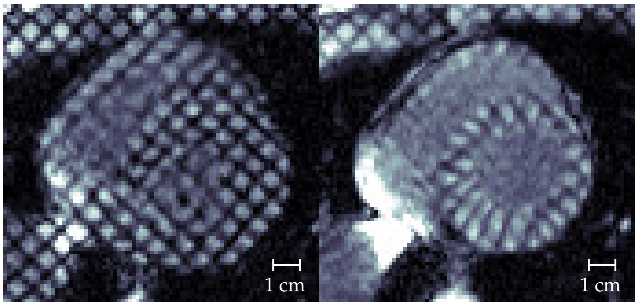

Fig. S3 A short-axis tagged cardiac MR imaging plane. The left panel shows the initial tagging near end-diastole, while the right panel illustrates myocardial deformations recorded at end-systole.

The myocardial displacements were registered from the tagged MRI data using a deformable image registration algorithm. The myocardial walls were outlined manually in each imaging plane before the image registration was performed. We described this image registration method previously in Hong (2018). This method registers LV displacements in each plane using a deformable mesh. Although tag fading exists, the registration of consecutive frames is largely unaffected as the intensity between consecutive images is reasonably consistent, despite substantial fading over the full time-course. These 2D registrations are combined to generate a 3D representation of the myocardial deformation using a cubic mesh. These registered displacements are illustrated in Figure S4.

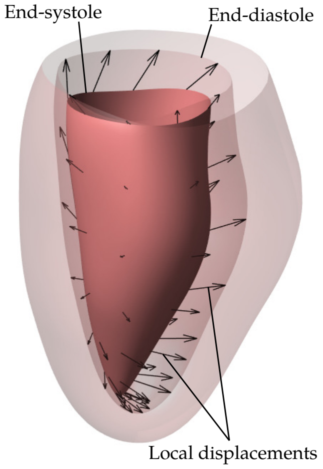

Fig. S4 Displacement vectors between end-systole and end-diastole at the endocardial wall registered from the tagged MRI. Vertical motion is registered using long-axis views while the torsion is registered with short-axis views. Greater expansion is seen at the lateral wall compared to the septal wall.

S7 Fiber direction basis vectors

In this section, we develop a method for defining fiber angles that vary linearly from the endocardium to the epicardium. The angles at the surfaces are typically chosen to match average values from the literature. We define the muscle fiber angles in the reference frame. The fiber angles are defined relative to continuous surfaces within the myocardial wall. These surfaces are defined in terms of an auxiliary variable

$$u = \frac{\mu_0 - \mu_{in0}}{\mu_{out0} - \mu_{in0}}, \tag{S29}$$

which has been constructed so that it is zero at the inner wall and one at the outer wall. The μ_0 coordinate is computed from u as

$$\mu_0 = (1-u)\mu_{in0}(\nu_0,\phi_0) + u\mu_{out0}(\nu_0,\phi_0). \tag{S30}$$

Internal surfaces are defined by constant values of u. The Cartesian coordinates of the internal surfaces are

$$\begin{aligned} x_0 &= a\sinh[\mu_0(u,\nu_0,\phi_0)]\sin\nu_0\cos\phi_0 \\ y_0 &= a\sinh[\mu_0(u,\nu_0,\phi_0)]\sin\nu_0\sin\phi_0 \\ z_0 &= a\cosh[\mu_0(u,\nu_0,\phi_0)]\cos\nu_0. \end{aligned} \tag{S31}$$

Surface tangent vectors are found by computing the cross product of the ν_0 and ϕ_0 derivatives:

$$\mathbf{w}_2 = \left[\frac{\partial x_0}{\partial \nu_0}, \frac{\partial y_0}{\partial \nu_0}, \frac{\partial z_0}{\partial \nu_0}\right] \quad \mathbf{w}_3 = \left[\frac{\partial x_0}{\partial \phi_0}, \frac{\partial y_0}{\partial \phi_0}, \frac{\partial z_0}{\partial \phi_0}\right]. \tag{S32}$$

Because u is a constant on these surfaces, the derivatives of μ_0 are

$$\begin{aligned} \frac{\partial \mu_0}{\partial \nu_0} &= (1-u)\frac{\partial \mu_{in0}}{\partial \nu_0} + u\frac{\partial \mu_{out0}}{\partial \nu_0} \\ \frac{\partial \mu_0}{\partial \phi_0} &= (1-u)\frac{\partial \mu_{in0}}{\partial \phi_0} + u\frac{\partial \mu_{out0}}{\partial \phi_0}, \end{aligned} \tag{S33}$$

where the partial derivatives of μ_{in0} and μ_{out0} are the exact derivatives of the bicubic functions. The local basis vectors are computed by dividing by the Euclidean norm:

$$\mathbf{e}_2 = \frac{\mathbf{w}_2}{|\mathbf{w}_2|} \quad \mathbf{e}_3 = \frac{\mathbf{w}_3}{|\mathbf{w}_3|}. \tag{S34}$$

Note that at the apex ($\nu = \pi$) $|\mathbf{w}_3| = 0$ and the expression above is singular. A suitable alternative expression at the apex is

$$\mathbf{e}_3 = [-\sin\phi_0, \cos\phi_0, 0]^\top. \tag{S35}$$

We construct a vector e_4 that lies in the x-y and $\mathbf{e}_2$-$\mathbf{e}_3$ planes. Rodrigues' formula gives a vector e_4 that has been rotated an angle θ about the vector e_1:

$$\begin{aligned} 0 &= ([\mathbf{e}_3]_3 - [\mathbf{e}_1]_3(\mathbf{e}_1\cdot\mathbf{e}_3))\cos\theta \\ &\quad + [\mathbf{e}_1\times\mathbf{e}_3]_3\sin\theta + [\mathbf{e}_1]_3(\mathbf{e}_1\cdot\mathbf{e}_3). \end{aligned} \tag{S36}$$

This condition has the form

$$0 = a\cos\theta + b\sin\theta + c. \tag{S37}$$

The solution is

$$\begin{aligned} \cos\theta &= -\frac{ac - \sqrt{b^2(a^2+b^2-c^2)}}{a^2+b^2} \\ \sin\theta &= -\frac{b^2c + a\sqrt{b^2(a^2+b^2-c^2)}}{b(a^2+b^2)}. \end{aligned} \tag{S38}$$

The resulting vector $\mathbf{e}_4$ lies in the x-y plane and is normal to the internal constant u surface. We define fiber coordinates and fiber angles relative to this direction.

We define fiber angles that vary linearly through the wall

$$\psi_1(w) = \psi_{in0} + (\psi_{out0} - \psi_{in0})w, \tag{S39}$$

where w increases from zero at the endocardium to one at the epicardium. A simple choice for w is

$$w(\nu_0,\phi_0) = \frac{\mu_0 - \mu_{in0}}{\mu_{out0} - \mu_{in0}}. \tag{S40}$$

Alternatively, w may be scaled according to the distance along lines of constant ν_0 and ϕ_0. The scale factor in the μ_0 direction is

$$g_{\mu_0} = a\sqrt{\sinh^2\mu_0 + \sin^2\nu_0}. \tag{S41}$$

Integrating the scale factor along these lines gives the distance:

$$L(\mu_0,\nu_0,\phi_0) = \int_{\mu_{in0}(\nu_0,\phi_0)}^{\mu_0} a\sqrt{\sinh^2 u + \sin^2\nu_0}\, du. \tag{S42}$$

We approximate this integral by the trapezoid rule because it lacks an analytical solution. Thus,

$$w(\nu_0,\phi_0) = \frac{L(\nu_0,\phi_0)}{L(\mu_{out0},\nu_0,\phi_0)}. \tag{S43}$$

We use the cutoff function (S15) defined in the appendix to send the fiber angles to 90° at the apex. Thus the final form of ψ is

$$\psi = \psi_1 + (1-G)[(\mathrm{sign}(\psi_1))\pi/2 - \psi_1]. \tag{S44}$$

The local fiber directions are defined in terms of the local fiber coordinates(s,n,f). The fiber direction is defined by rotating the reference vector $\mathbf{e}_4$ by an angle ψ in the surface tangent plane. The fiber direction is

$$\mathbf{e}_f = \mathbf{e}_4\cos\psi + (\mathbf{e}_1\times\mathbf{e}_4)\sin\psi + \mathbf{e}_1(\mathbf{e}_1\cdot\mathbf{e}_4)(1-\cos\psi). \tag{S45}$$

The s coordinate direction is the surface normal vector, i.e.,

$$\mathbf{e}_s = \mathbf{e}_1. \tag{S46}$$

To generate a right handed orthogonal coordinate system, the final fiber coordinate direction is

$$\mathbf{e}_n = \mathbf{e}_f \times \mathbf{e}_s. \tag{S47}$$

S8 Surface tangent vectors

The endocardial surface tangent vectors (in the deformed configuration) are

$$\mathbf{v}_{e1} = \left[\frac{\partial x}{\partial\nu_0}, \frac{\partial y}{\partial\nu_0}, \frac{\partial z}{\partial\nu_0}\right] \quad \mathbf{v}_{e2} = \left[\frac{\partial x}{\partial\phi_0}, \frac{\partial y}{\partial\phi_0}, \frac{\partial z}{\partial\phi_0}\right]. \tag{S48}$$

These derivatives are

$$\begin{aligned}
\frac{\partial x}{\partial\theta_0} &= a\left(\cosh\mu\sin\nu\cos\phi\frac{\partial\mu}{\partial\theta_0} + \sinh\mu\cos\nu\cos\phi\frac{\partial\nu}{\partial\theta_0}\right.\\
&\qquad\left. - \sinh\mu\sin\nu\sin\phi\frac{\partial\phi}{\partial\theta_0}\right)\\
\frac{\partial y}{\partial\theta_0} &= a\left(\cosh\mu\sin\nu\sin\phi\frac{\partial\mu}{\partial\theta_0} + \sinh\mu\cos\nu\sin\phi\frac{\partial\nu}{\partial\theta_0}\right.\\
&\qquad\left. + \sinh\mu\sin\nu\cos\phi\frac{\partial\phi}{\partial\theta_0}\right)\\
\frac{\partial z}{\partial\theta_0} &= a\left(\sinh\mu\cos\nu\frac{\partial\mu}{\partial\theta_0} - \cosh\mu\sin\nu\frac{\partial\nu}{\partial\theta_0}\right),
\end{aligned} \tag{S49}$$

where for $\mathbf{v}_{e1}$: $\theta_0 = \nu_0$ and for $\mathbf{v}_{e2}$: $\theta_0 = \phi_0$.

S9 Closing surface Γ

The LV chamber must be closed to define the volume of the cavity and approximate work done at the base of the LV. However, an appropriate lid is difficult to define in prolate spheroidal coordinates. As a result, we define the lid in cylindrical coordinates.

S9.1 Cavity volume

The cavity volume is computed with three terms. This is necessary because of singularities associated with the prolate coordinate system at the axis.

$$V_{lv} = V_1 + V_2 + V_3. \tag{S50}$$

V_1 is computed as

$$\begin{aligned}
V_1 = a^3\int_0^{2\pi}\int_{\nu_{up}(\phi_0)}^{\pi} \sin\nu\left[\frac{1}{3}\left(\cosh^3\mu_{in} - 1\right)\right.\\
\left. + \cos^2\nu(1-\cosh\mu_{in})\right] K\, d\nu_0 d\phi_0.
\end{aligned} \tag{S51}$$

Another volume is computed between the boundary of the first volume at the base and a second surface that has a fixed ν value at the axis. For simplicity we set the value of ν at the axis to ν_{up0}, the largest value of ν_{up} in the reference configuration. This choice is unimportant because it does not define the final closing surface. This surface is defined in the prolate coordinate system. The μ values are

$$\mu(\mu_0,\phi_0) = \frac{\mu_{endo}(\phi_0)}{\mu_{0,endo}(\phi_0)}\mu_0. \tag{S52}$$

The ν values are

$$\nu(\mu_0,\phi_0) = \nu_{avg} + (\nu_{up}(\phi_0) - \nu_{avg})\frac{\mu_0}{\mu_{0,endo}(\phi_0)}. \tag{S53}$$

The volume V_2 is therefore

$$V_2 = \frac{a^3}{6}\int_0^{2\pi}\int_0^{\mu_{in}(\nu_{up}(\phi_0),\phi_0)} \cos\nu_{up}\sinh\mu\,[\cos(2\nu_{up}) - 3\cosh(2\mu)-2] - \cos\nu_l \sinh\mu\,[\cos(2\nu_{R_2}) - 3\cosh(2\mu)-2]\,d\mu d\phi. \tag{S54}$$

We define a third surface that determines the actual boundary of the LV cavity at the base. This surface is defined in cylindrical coordinates to avoid singularities associated with prolate spheroidal coordinate system surfaces. The cylindrical coordinates of this boundary can be expressed as

$$\begin{aligned} r_{out}(\phi_0) &= a\sinh(\mu(\phi_0))\sin(\nu(\phi_0)) \\ z_{out}(\phi_0) &= a\cosh(\mu(\phi_0))\cos(\nu(\phi_0)). \end{aligned} \tag{S55}$$

The surface is defined on the domain $u_r \in [0,1]\times\phi_0 \in [0,2\pi)$. The value of the radius is given in terms of the reduced radial coordinate u_r by

$$r(\phi_0) = u_r r_{out}(\phi_0). \tag{S56}$$

The z-coordinate is defined through

$$z_{lid} = (1-u_r)z_{mean} + u_r z_{out}(\phi_0). \tag{S57}$$

The value of z_{mean} is the value of z at the axis. This value is averaged from the endocardial z values as

$$z_{mean} = \frac{1}{2\pi}\int_0^{2\pi} z_{out}(\phi_0)\frac{\partial\phi}{\partial\phi_0}d\phi_0. \tag{S58}$$

This choice provides a nearly flat closing surface for the cavity volume. The third volume term is therefore

$$V_3 = \int_0^{2\pi}\int_0^1 u_r(z_{lid}-z_2)r_{out}^2 du_r \frac{\partial\phi}{\partial\phi_0}d\phi, \tag{S59}$$

where z_2 is the z value of the previous surface at the same (r,ϕ) locations.

S9.2 Basal work

Conservation of virtual work requires a term for the work done at the base. The basal work is equal to the work done by the fluid through the chamber closing surface Γ. The traction vector is

$$\mathbf{T} = P_{lv}\mathbf{n}. \tag{S60}$$

We define surface tangent vectors in terms of the Cartesian coordinate vector

$$\mathbf{r}_l(u_r,\phi_0) = [x(u_r,\phi_0), y(u_r,\phi_0), z(u_r,\phi_0)]. \tag{S61}$$

The local surface tangent vectors are

$$\mathbf{v}_{l1} = \left[\frac{\partial x}{\partial u_r},\frac{\partial y}{\partial u_r},\frac{\partial z}{\partial u_r}\right] \quad \mathbf{v}_{l2} = \left[\frac{\partial x}{\partial\phi_0},\frac{\partial y}{\partial\phi_0},\frac{\partial z}{\partial\phi_0}\right]. \tag{S62}$$

The surface normal can be computed as the normalized cross product of the two tangent surface vectors:

$$\mathbf{n} = \frac{\mathbf{v}_{l1}\times\mathbf{v}_{l2}}{|\mathbf{v}_{l1}\times\mathbf{v}_{l2}|}. \tag{S63}$$

The partial derivatives are

$$\begin{aligned} \frac{\partial x}{\partial u_r} &= r_{out}(\phi_0)\cos(\phi(\phi_0)) \\ \frac{\partial x}{\partial\phi_0} &= u_r\frac{\partial r_{out}}{\partial\phi_0}\cos\phi - r\sin\phi\frac{\partial\phi}{\partial\phi_0} \\ \frac{\partial y}{\partial u_r} &= r_{out}(\phi_0)\sin(\phi(\phi_0)) \\ \frac{\partial y}{\partial\phi_0} &= u_r\frac{\partial r_{out}}{\partial\phi_0}\sin\phi + r\cos\phi\frac{\partial\phi}{\partial\phi_0} \\ \frac{\partial z}{\partial u_r} &= -z_{mean} + z_{out}(\phi_0) \\ \frac{\partial z}{\partial\phi_0} &= u_r\frac{\partial z_{out}}{\partial\phi_0}, \end{aligned} \tag{S64}$$

where the two auxiliary derivatives are

$$\begin{aligned} \frac{\partial r_{out}}{\partial\phi_0} =& a\sin\nu_{out}\cosh\mu_{out}\frac{\partial\mu_{out}}{\partial\phi_0} + \\ & a\cos\nu_{out}\sinh\mu_{out}\frac{\partial\nu_{out}}{\partial\phi_0} \\ \frac{\partial z_{out}}{\partial\phi_0} =& a\cos\nu_{out}\sinh\mu_{out}\frac{\partial\mu_{out}}{\partial\phi_0} \\ & - a\sin\nu_{out}\cosh\mu_{out}\frac{\partial\nu_{out}}{\partial\phi_0}. \end{aligned} \tag{S65}$$

S10 Lumped parameter system

The lumped parameter system includes variable resistance mitral and aortic valves along with 0D lumped parameter models for the left atrium, systemic arteries, and systemic periphery. The circulation is not treated as a closed loop, but is terminated with fixed pressures P_{pv} and P_{sv} at the pulmonary and systemic venous systems, respectively. The variable resistance valves are described by

$$R_{mv} = R_{mv,c} - \frac{R_{mv,c}-R_{mv,o}}{1+e^{-\beta(P_{la}-P_{lv})}} \tag{S66}$$

$$R_{aov} = R_{aov,c} - \frac{R_{aov,c}-R_{aov,o}}{1+e^{-\beta(P_{lv}-P_{pao})}}. \tag{S67}$$

The parameters are defined in Table S2. P_{la} and P_{pao} represent the pressures in the left atrium and proximal aorta, respectively. The lumped parameter models are described by

a set of volume-conservation ODEs.

$$\begin{aligned}\frac{dP_{la}}{dt} &= \frac{1}{C_{la}}\left(\frac{P_{pv}-P_{la}}{R_{pv}} - \frac{P_{la}-P_{lv}}{R_{mv}}\right)\\ \frac{dP_{sa}}{dt} &= \frac{1}{C_{sa}}\left(\frac{P_{pao}-P_{sa}}{R_{pao}} - \frac{P_{sa}-P_{sp}}{R_{sa}}\right)\\ \frac{dP_{sp}}{dt} &= \frac{1}{C_{sp}}\left(\frac{P_{sa}-P_{sp}}{R_{sa}} - \frac{P_{sp}-P_{sv}}{R_{sp}}\right)\end{aligned} \tag{S68}$$

The compliances, resistances, and fixed pressures are defined in Table S2. The variables P_{sa} and P_{sp} represent the pressure in the systemic arteries and systemic periphery, respectively. One additional algebraic equation is included to model the resistance of the proximal aorta:

$$P_{pao}(R_{pao}+R_{aov}) - P_{lv}R_{pao} - P_{sa}R_{aov} = 0. \tag{S69}$$

S11 Numerical solution to the coupled system

Combining the LV ODE system (41), LV blood volume conservation (42), and proximal aorta pressure drop (S69), yields the coupled system:

$$\begin{aligned}0 &= \alpha_{1,1}\frac{dq_1}{dt} + \ldots + \alpha_{1,N_q}\frac{dq_{N_q}}{dt} + \kappa_1 - \eta_1 P_{lv}\\ 0 &= \alpha_{2,1}\frac{dq_1}{dt} + \ldots + \alpha_{2,N_q}\frac{dq_{N_q}}{\partial t} + \kappa_2 - \eta_2 P_{lv}\\ &\vdots \qquad\qquad \vdots\\ 0 &= \alpha_{N_q,1}\frac{dq_1}{dt} + \ldots + \alpha_{N_q,N_q}\frac{dq_{N_q}}{dt} + \kappa_{N_q} - \eta_{N_q} P_{lv}\\ 0 &= \frac{\partial V}{\partial q_1}\frac{dq_1}{dt} + \ldots + \frac{\partial V}{\partial q_{n_q}}\frac{dq_{n_q}}{dt} - \frac{P_{la}-P_{lv}}{R_{mv}} + \frac{P_{lv}-P_{pao}}{R_{aov}}\\ 0 &= P_{pao}(R_{pao}+R_{aov}) - P_{lv}R_{pao} - P_{sa}R_{aov}.\end{aligned} \tag{S70}$$

This is a square $(n_q+2)\times(n_q+2)$ system. It is linear in all terms except R_{mv} and R_{aov}, which depend exponentially on the pressures in the left ventricle P_{lv} and proximal aorta P_{pao}. We solve this system for the time derivatives of the LV deformation parameters, LV pressure, and proximal aorta pressure using a Newton iteration. We define the residual function $\boldsymbol{H}$ to be the right hand side of the coupled system (S70). Defining the Newton iteration variable

$$\boldsymbol{X} = \left[\frac{dq_1}{dt}, \ldots, \frac{dq_{N_q}}{dt}, P_{lv}, P_{pao}\right]^\top, \tag{S71}$$

the Jacobian is

$$\boldsymbol{J}(\boldsymbol{X}) = \begin{bmatrix} \alpha_{1,1} & \alpha_{1,2} & \cdots & \alpha_{1,n_q} & -\eta_1 & 0\\ \alpha_{2,1} & \alpha_{2,2} & \cdots & \alpha_{2,n_q} & -\eta_2 & 0\\ \vdots & & & \vdots & & \\ \alpha_{n_q,1} & \alpha_{n_q,2} & \cdots & \alpha_{n_q,n_q} & -\eta_{n_q} & 0\\ \frac{\partial V}{\partial q_1} & \frac{\partial V}{\partial q_2} & \cdots & \frac{\partial V}{\partial q_{n_q}} & \frac{\partial H_{N_q+1}}{\partial P_{lv}} & \frac{\partial H_{N_q+1}}{\partial P_{pao}}\\ 0 & 0 & \cdots & 0 & \frac{\partial H_{N_q+2}}{\partial P_{lv}} & \frac{\partial H_{N_q+2}}{\partial P_{pao}} \end{bmatrix}. \tag{S72}$$

The derivatives required by the Jacobian may be computed exactly using

$$\begin{aligned}\frac{\partial R_{mv}}{\partial P_{lv}} &= \frac{\beta(R_{mv,c}-R_{mv,o})}{\left(e^{\frac{1}{2}\beta(P_{la}-P_{lv})} + e^{-\frac{1}{2}\beta(P_{la}-P_{lv})}\right)^2}\\ \frac{\partial R_{aov}}{\partial P_{lv}} &= \frac{-\beta(R_{aov,c}-R_{aov,o})}{\left(e^{\frac{1}{2}\beta(P_{lv}-P_{pao})} + e^{-\frac{1}{2}\beta(P_{lv}-P_{pao})}\right)^2}\\ \frac{\partial R_{aov}}{\partial P_{pao}} &= \frac{\beta(R_{aov,c}-R_{aov,o})}{\left(e^{\frac{1}{2}\beta(P_{lv}-P_{pao})} + e^{-\frac{1}{2}\beta(P_{lv}-P_{pao})}\right)^2}.\end{aligned} \tag{S73}$$

These equations have been written in a form that avoids numerical precision errors in the exponentials. The Jacobian entries are consequently

$$\begin{aligned}\frac{\partial H_{N_q+1}}{\partial P_{lv}} &= \frac{R_{mv} + (P_{la}-P_{lv})\frac{\partial R_{mv}}{\partial P_{lv}}}{{R_{mv}}^2}\\ &\quad + \frac{R_{aov} - (P_{lv}-P_{pao})\frac{\partial R_{aov}}{\partial P_{lv}}}{{R_{aov}}^2}\\ \frac{\partial H_{N_q+1}}{\partial P_{pao}} &= -\frac{R_{aov} + (P_{lv}-P_{pao})\frac{\partial R_{aov}}{\partial P_{pao}}}{{R_{aov}}^2}\\ \frac{\partial H_{N_q+2}}{\partial P_{lv}} &= P_{pao}\frac{\partial R_{aov}}{\partial P_{lv}} - R_{pao} - P_{sa}\frac{\partial R_{aov}}{\partial P_{lv}}\\ \frac{\partial H_{N_q+2}}{\partial P_{pao}} &= R_{pao} + R_{aov} + P_{pao}\frac{\partial R_{aov}}{\partial P_{pao}} - P_{sa}\frac{\partial R_{aov}}{\partial P_{pao}}.\end{aligned} \tag{S74}$$

A Newton iteration updates the vector $\boldsymbol{X}$ by solving

$$\boldsymbol{J}(\boldsymbol{X}_i)(\boldsymbol{X}_{i+1} - \boldsymbol{X}_i) = -\boldsymbol{H}(\boldsymbol{X}_i) \tag{S75}$$

for $\boldsymbol{X}_{i+1}$ and iterating to convergence. The full ODE system includes the time derivatives of the kinematic variables $\boldsymbol{q}$ which were solved for in the Newton interation and those of the lumped parameter models (S68). This ODE system is solved using a standard explicit time stepping scheme such as 4th order Runge-Kutta (RK4).

S12 Analytic cylinder solution

The stress distributions evaluated by the prolate spheroidal model are compared to the analytic cylinder stress distributions in Figure S5. The isotropic component of stress σ_{iso} is not computed in the virtual work equations, but is integrated numerically from the computed solution.

Following the analysis of Rivlin (1949), using the convenient formulation written by Nash (1998), we consider an infinitely extended hollow incompressible cylinder of an isotropic Mooney-Rivlin material. The deviatoric part of the elastic strain energy is:

$$W = c_1(I_1 - 3) + c_2(I_2 - 3), \tag{S76}$$

where the invariants are

$$I_1 = \mathrm{tr}\boldsymbol{C} \quad \text{and} \quad I_2 = \frac{1}{2}\left[(\mathrm{tr}\boldsymbol{C})^2 - \mathrm{tr}\boldsymbol{C}^2\right]. \tag{S77}$$

The elastic component of the PK2 stress $\boldsymbol{S}_e$ is computed from the elastic strain energy (S76) using (26). The cylinder is assumed to have undergone a fixed stretch λ in the z direction as well as a fixed torsion ψ. In cylindrical coordinates (r,θ,z), the deformation is

$$r = r(r_0) \qquad \theta = \theta_0 + \psi\lambda z_0 \qquad z = \lambda z_0. \tag{S78}$$

Rivlin solved the finite elasticity problem under pressure loading conditions p_{in} on the interior surface and p_{ext} at the exterior surface. The radial deformation $r(r_0)$ is computed by solving the strong form of the equilibrium equations (44). The results are outlined here for reference.

$$\mu(r_0) = \sqrt{\frac{1}{\lambda}\left(1 + \frac{K}{r_0^2}\right)}, \tag{S79}$$

where

$$K = a_{in}^2(\lambda\mu_{in}^2 - 1) = a_{out}^2(\lambda\mu_{out}^2 - 1). \tag{S80}$$

Here a_{in} is the undeformed inner radius, a_{out} the undeformed outer radius, $\mu_{in} = \mu(a_{in})$, and $\mu_{out} = \mu(a_{out})$. The analytical Cauchy stress components are

$$\begin{aligned}
\sigma_{rr} &= 2\left[\frac{c_1}{\lambda^2\mu^2} + c_2\left(\frac{1}{\lambda^2} + \frac{1}{\mu^2} + \psi^2 r_0^2\right)\right] - \sigma_{iso}\\
\sigma_{\theta\theta} &= 2\left[c_1\mu^2(1 + \psi^2\lambda^2 r_0^2) + c_2\left(\frac{1}{\lambda^2} + \lambda^2\mu^2 + \psi^2 r_0^2\right)\right]\\
&\quad -\sigma_{iso}\\
\sigma_{zz} &= 2\left[c_1\lambda^2 + c_2\left(\frac{1}{\mu^2} + \lambda^2\mu^2\right)\right] - \sigma_{iso}\\
\sigma_{rz} &= \sigma_{r\theta} = 0\\
\sigma_{\theta z} &= 2\left[c_1\psi\lambda^2\mu r_0 + \frac{c_2\psi r_0}{\mu}\right]
\end{aligned} \tag{S81}$$

with the isotropic component of the stress given by

$$\begin{aligned}
\sigma_{iso} = -p_{out} &+ \left(\frac{c_1}{\lambda} + c_2\lambda\right)\left[\frac{1}{\lambda\mu_{out}^2} - \frac{r_0^2}{r_0^2 + K} + \ln\left(\frac{\mu^2}{\mu_{out}^2}\right)\right]\\
&- c_1\psi^2\lambda(r_0^2 - a_{out}^2) + 2\left[\frac{c_1}{\lambda^2\mu^2} + c_2\left(\frac{1}{\lambda^2} + \frac{1}{\mu^2} + \psi^2 r_0^2\right)\right].
\end{aligned} \tag{S82}$$

The system may be solved by first numerically solving for μ_{out} using the boundary condition

$$\sigma_{rr}(a_{in}) = -p_{in} \tag{S83}$$

and subsequently solving for μ_{in} in (S80). The remainder of the variables are evaluated explicitly.

To compare our model solution to the analytical solution, we set the focal length of the prolate spheroidal coordinate system to $a = 100$, which produces an extremely elongated ellipsoid. In this configuration, the equatorial region closely approximates a cylinder. We limit the ν coordinate to include only a 1 cm region near the equator, and solve the virtual work equations using the Mooney-Rivlin material law. For this simulation, no active or viscous stresses are assumed, so the total stress (38) is given by $\boldsymbol{S} = \boldsymbol{S}_e$. At the interior and exterior surfaces, the boundary conditions are as for the analytic solution, while natural boundary conditions are used at the other surfaces. The equilibrium virtual work system (43) is solved for the deformation with the assumed cavity pressure $P_{lv} = p_{in}$. We use the same parameters as those used by Nash (1998) shown in Table S3.

Table S3 Analytical cylinder parameters.

Parameter	Description	Value
a_{in}	Internal radius	1.0 cm
a_{out}	External radius	1.5 cm
c_1	Material parameter	2.0 kPa
c_2	Material parameter	6.0 kPa
ψ	Fixed torsion	30°/cm
λ	Fixed extension ratio	1.2
p_{in}	Internal pressure	1.5 kPa
p_{ext}	External pressure	0 kPa

The stress distributions evaluated by the prolate spheroidal model are compared to the analytic cylinder stress distributions in Figure S5. The isotropic component of stress p is not computed in the virtual work equations, but is integrated numerically from the computed solution.

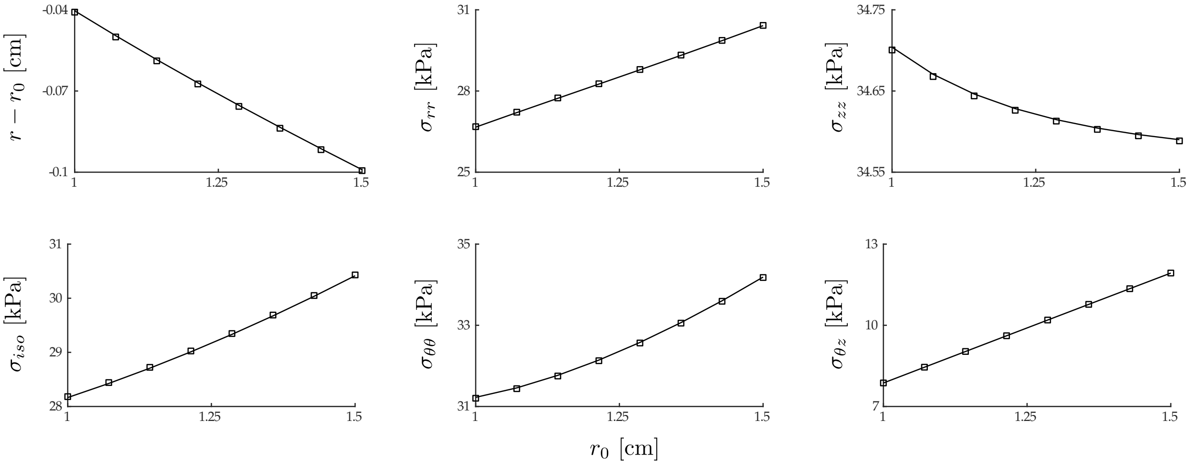

Fig. S5 Comparison between the analytic cylinder solution (–) and the proposed model solution (□) using the parameter values in Table S3. σ_{iso} is the isotropic stress component that results from the material incompressibility.

S13 Cauchy stress divergence

We compute the Cauchy stress divergence in Cartesian coordinates. The Cartesian representation of the Cauchy stress may be computed from the prolate coordinate representation of the PK2 stress using

$$[\boldsymbol{\sigma}]_{cart} = \frac{1}{J}\boldsymbol{Q}[\boldsymbol{F}]_{prol}[\boldsymbol{S}]_{prol}[\boldsymbol{F}^\top]_{prol}\boldsymbol{Q}^\top, \tag{S84}$$

where $\boldsymbol{Q}$ is a rotation matrix that transforms from prolate to Cartesian coordinates. This matrix is defined analogously to (22) as

$$\boldsymbol{Q} = \begin{bmatrix} \boldsymbol{e}_x \cdot \boldsymbol{e}_\mu & \boldsymbol{e}_x \cdot \boldsymbol{e}_\nu & \boldsymbol{e}_x \cdot \boldsymbol{e}_\phi \\ \boldsymbol{e}_y \cdot \boldsymbol{e}_\mu & \boldsymbol{e}_y \cdot \boldsymbol{e}_\nu & \boldsymbol{e}_y \cdot \boldsymbol{e}_\phi \\ \boldsymbol{e}_z \cdot \boldsymbol{e}_\mu & \boldsymbol{e}_z \cdot \boldsymbol{e}_\nu & \boldsymbol{e}_z \cdot \boldsymbol{e}_\phi \end{bmatrix}. \tag{S85}$$

To approximate derivatives of the Cartesian Cauchy stress, we first approximate derivatives with respect to the undeformed prolate spheroidal coordinates $\boldsymbol{\Theta} = [\Theta_1, \Theta_2, \Theta_3] = [\mu_0, \nu_0, \phi_0]$ using finite differences. For example:

$$\frac{\partial[\boldsymbol{\sigma}]_{cart}}{\partial\Theta_1} = \frac{[\boldsymbol{\sigma}]_{cart}(\Theta_1 + \Delta\Theta_1, \Theta_2, \Theta_3) - [\boldsymbol{\sigma}]_{cart}(\Theta_1, \Theta_2, \Theta_3)}{\Delta\Theta_1} \tag{S86}$$

approximates the derivative with respect to the first prolate spheroidal coordinate. These may be converted to derivatives with respect to the deformed Cauchy stress using

$$\frac{\partial[\boldsymbol{\sigma}]_{cart}}{\partial x_i} = \frac{\partial[\boldsymbol{\sigma}]_{cart}}{\partial\Theta_j}\frac{\partial\Theta_j}{\partial x_i}. \tag{S87}$$

To this end, we define the tensor transformation $\boldsymbol{T}$ by

$$T_{ij} = \frac{\partial x_i}{\partial\Theta_j} = \frac{\partial x_i}{\partial\theta_k}\frac{\partial\theta_k}{\partial\Theta_j}, \tag{S88}$$

where θ are the deformed prolate spheroidal coordinates. Note that

$$[T^{-1}]_{ij} = \frac{\partial\Theta_i}{\partial x_j}, \tag{S89}$$

implying that we may compute the Cartesian divergence using

$$\frac{\partial[\boldsymbol{\sigma}]_{cart}}{\partial x_i} = \frac{\partial[\boldsymbol{\sigma}]_{cart}}{\partial\Theta_j}[T^{-1}]_{ij}. \tag{S90}$$

The matrices required to compute $\boldsymbol{T}$ are

$$\frac{\partial\boldsymbol{x}}{\partial\boldsymbol{\theta}} = a\begin{bmatrix} \cosh\mu\sin\nu\cos\phi & \sinh\mu\cos\nu\cos\phi & -\sinh\mu\sin\nu\sin\phi \\ \cosh\mu\sin\nu\sin\phi & \sinh\mu\cos\nu\sin\phi & \sinh\mu\sin\nu\cos\phi \\ \sinh\mu\cos\nu & -\cosh\mu\sin\nu & 0 \end{bmatrix} \tag{S91}$$

and

$$\frac{\partial\boldsymbol{\theta}}{\partial\boldsymbol{\Theta}} = \begin{bmatrix} \frac{\partial\mu}{\partial\mu_0} & \frac{\partial\mu}{\partial\nu_0} & \frac{\partial\mu}{\partial\phi_0} \\ \frac{\partial\nu}{\partial\mu_0} & \frac{\partial\nu}{\partial\nu_0} & \frac{\partial\nu}{\partial\phi_0} \\ \frac{\partial\phi}{\partial\mu_0} & \frac{\partial\phi}{\partial\nu_0} & \frac{\partial\phi}{\partial\phi_0} \end{bmatrix}. \tag{S92}$$
